# Prognostic factors for change in memory test performance after memory training in healthy older adults: a systematic review and outline of statistical challenges

**DOI:** 10.1186/s41512-020-0071-8

**Published:** 2020-05-21

**Authors:** Mandy Roheger, Ann-Kristin Folkerts, Fabian Krohm, Nicole Skoetz, Elke Kalbe

**Affiliations:** 1 Department of Medical Psychology | Neuropsychology and Gender Studies & Center for Neuropsychological Diagnostics and Intervention (CeNDI), Faculty of Medicine and University Hospital Cologne, University of Cologne, Kerpener Str. 68, 50937 Cologne, Germany; 2 Evidence-Based Oncology, Department I of Internal Medicine, Faculty of Medicine and University Hospital Cologne, University of Cologne, Kerpener Str. 62, 50937 Cologne, Germany

**Keywords:** Prognostic factors, Memory training, Prediction, Verbal memory

## Abstract

**Background:**

The goal is to investigate prognostic factors for change in memory test performance in healthy older adults and to report and discuss the different statistical procedures used for investigating this topic in the literature.

**Methods:**

Prognostic factors were here understood as any measures that were investigated to estimate change in memory test performance. MEDLINE, Web of Science Core Collection, CENTRAL, and PsycInfo were searched up to November 2019. Prognostic factor and prognostic factor finding studies investigating prognostic factors on verbal and non-verbal short- and long-term memory after conducting memory training in healthy older adults were included. Risk of bias was assessed using the QUIPS tool.

**Results:**

Our search yielded 12,974 results. We included 29 studies that address prognostic factors of change in memory test performance, including sociodemographic, (neuro-)psychological, genetic, and biological parameters. Studies showed high variation and methodological shortcomings with regard to the assessment, statistical evaluation, and reporting of the investigated prognostic factors. Included studies used different types of dependent variables (change scores vs. post-test scores) when defining change in memory test performance leading to contradictory results. Age was the only variable investigated throughout most of the studies, showing that older adults benefit more from training when using the change score as the dependent variable.

**Conclusion:**

Overall, there is a need for adequate reporting in studies of prognostic factors for change in memory test performance. Because of inconsistencies and methodological shortcomings in the literature, conclusions regarding prognostic factors remain uncertain. As a tentative conclusion, one may say that the higher the age of the participant, the more profound the improvement in memory test performance will be after memory training.

**Trial registration:**

CRD42019127479.

## Background

Even in the absence of severe health issues, the aging process is associated with a decline in cognitive functioning, e.g., in memory, attention, or executive functions, which may result in a loss of autonomy and quality of life in older individuals [1]. One way that has been discussed to be able to contribute to maintenance of cognitive function in the older age (> 55 years) is cognitive training (CT, defined as guided cognitive exercises designed to improve specific cognitive functions, as well as enhance performance in untrained cognitive tasks [2]). Recent meta-analyses and reviews show that CT can be effective not only in improving cognitive functions in healthy older individuals, but also their quality of life [3, 4]. There are many different types of CT, which differ regarding their settings (e.g., single vs. group settings), materials used (e.g., computerized vs. paper-and-pencil tasks), but also regarding their focus on different outcomes (e.g., memory, attention, executive functions). Memory, which is a key function that typically decreases in higher age, even in healthy older adults [5], can also be improved or maintained with the help of CT [4]. However, one question that remains under-investigated is: who (with which profile of, e.g., sociodemographic, neuropsychological, genetic parameters) benefits from CT? Yet, identifying prognostic factors is highly important for providing new treatment options and in term of dementia prevention [6]. Prognostic factors (in literature also often referred to as “predictors”) for changes in test performance after a CT that are under debate are sociodemographic variables, brain imaging parameters, genetic parameters, and blood factors, as well as personality traits, cognitive and non-cognitive abilities at the entry of the training, and different training characteristics, e.g., intensity of the trainings [7]. Yet, data is highly inconsistent: for example, there are several studies that report higher age as a positive prognostic factor for changes in test performance after a CT in healthy older adults [7, 8], while some studies indicate that younger individuals show improvement in test performance after a CT [9, 10].

Yet, inconsistent results regarding prognostic factors of CT can be seen throughout the prognostic factor literature for CT benefits so far, and the question arises, why this is the case. Until now, no systematic review exists investigating prognostic factors for CT success in healthy older adults in general, and memory training in particular to answer this question [11]. However, considering the fact that prognostic factors for change in cognitive performances after a CT in healthy older adults have many potential uses (e.g., aiding treatment and lifestyle decisions, improving individual dementia risk prediction, providing new treatment options [6]), and data so far reveals highly inconsistent results, systematic reviews and meta-analyses are urgently needed to summarize evidence about the prognostic value of particular factors to help to match cognitive interventions to individuals to improve their effectiveness in regard of a personalized medicine approach [12, 13].

Therefore, the present review focuses on prognostic factors for changes in memory performances after memory training, due to different reasons: first, memory belongs to the most vulnerable cognitive functions in aging (e.g., [5]). Second, we wanted to get a first overview over the published data on prognostic research after training interventions in a narrower frame, therefore focusing only on one specific relevant domain. Conclusions from this review could then help further research on prognostic factors of cognitive change induced by CTs.

### Objectives

The main goal of the present systematic review is to investigate prognostic factors for changes in memory performance after memory training in healthy older adults. Further, we wanted to investigate different methods used to evaluate prognostic factors for changes in memory performance after memory training. Based on the checklist for critical appraisal and data extraction for systematic reviews of prediction modelling studies [12, 14, 15], which can also be used to assess prognostic factors studies [12], we defined our systematic review question using the “PICOTS system” [15]. Our target population are healthy older individuals, defined as individuals aged ≥ 55 years with absence of any neurological or psychiatric disease (P). Regarding the investigated intervention (I), we investigated all prognostic factors assessed for change in memory test performance after memory training. No comparator factor is being considered (C). Outcome events for this review are changes in memory test performance after memory training in the domains verbal short-term memory, verbal long-term memory, as well as non-verbal short- and long-term memory operationalized with objective and standardized measurement instruments (O). The measurement of the prognostic factor had to be done before the memory training started and all follow-up information on the outcomes (all time periods) was extracted from the studies (T). Finally, prognostic factor measurement was studied in non-clinical settings to provide prognostic information for possibilities of prevention of cognitive decline (in other words, possibilities to strengthen cognitive function) in cognitively intact individuals (S).

## Methods

The present systematic review was preregistered; the review protocol can be assessed at www.crd.york.ac.uk/PROSPERO/ (ID: CRD42019127479). The reporting follows the Preferred Reporting Items for Systematic Reviews and Meta-Analyses (PRISMA) guideline for systematic reviews and meta-analysis [16]. “The PRISMA for Abstracts Checklists”, as well as “The PRISMA checklist for systematic reviews” are displayed in Supplementary Tables 1 and 2.

### Search and study selection

A systematic search was conducted in MEDLINE Ovid, Web of Science Core Collection, CENTRAL, and PsycInfo up to October 2018. An update-search was conducted in the same data bases until 12th November 2019. Reference lists of all identified trials, relevant review articles, and current treatment guidelines were hand searched for further literature. In cases where no full text could be obtained, we contacted the authors and asked them to provide full text publications within a 2-week time frame. Further information on the systematic search and the full search strings for each database are presented in the Supplementary Material, Tables 3, 4, 5 and 6.

Titles and abstracts were screened according to predefined eligibility criteria by two individual review authors (MR and AKF) with the Covidence Software (Veritas Health Innovation) [17]. Afterwards, the full-text articles of the studies meeting the inclusion criteria were further reviewed for inclusion in the systematic review. In cases where no consensus could be reached between the two authors MR and AKF, a third author (NS) was asked and the case was discussed until a final consensus was reached.

### Eligibility criteria

The review focused on peer-reviewed studies in English and German with no limitations regarding publication date which investigated prognostic factors of changes in memory test performance after memory training. Full study reports needed to be available; abstracts, books, book chapters, study protocols, and conference papers were excluded.

Prognostic factor studies on healthy older participants (age ≥ 55 years) were included. Data from participants with dementia diagnosis, neurological and/or psychiatric diseases, as well as uncorrected seeing or hearing impairments, assessed at least via self-report, were excluded. Studies with participants with mild cognitive impairment (if reported) were also excluded as we want to investigate healthy adults in the context of interventions.

Regarding the investigated intervention and included prognostic factors, all prognostic factors (e.g., sociodemographic factors, brain imaging parameters, genetic parameters, blood factors, personality traits, cognitive abilities at the entry of the training, different training characteristics, e.g., intensity of the trainings, etc.) which investigate changes in memory test performance after memory training were included in the review and meta-analysis. Memory training was defined as a CT that targets primarily on memory performance with a minimum of two sessions in total. The memory training can either include computerized or paper-pencil tasks with clear cognitive rationale, which are administered either on personal devices or in individual- or group settings held by a facilitator. When multi-domain approaches were examined, memory had to be the main component of the program (at least 50% of the exercises).

Prognostic factor studies, which investigate memory training benefits as an outcome (verbal or non-verbal short- or long-term memory) measured with established objective neuropsychological tests, were included. Working memory was excluded and is being investigated in a different review, as we define working memory as an executive function rather than a pure memory function [18]. We excluded subjective self-rated memory scales, as well as measures of memory strategy use. The factor measurement of the included studies had to be conducted before the memory training started, and there was no limitation regarding the length of the follow-ups.

### Data extraction

Two review authors (MR and AKF) independently extracted the data according to the Critical appraisal and data extraction for systematic reviews of prediction modelling studies_ prognostic factors (CHARMS_PF) checklist [15] to investigate the reporting of prognostic factors.

### Quality assessment

Two reviewers (MR and AKF) independently assessed the extracted studies for the risk of bias using the Quality in Prognosis Studies (QUIPS) checklist, developed by Hayden et al. [19] to examine the risk of bias in prognostic factors studies across six domains [19]: Study participation, study attrition, prognostic factor measurement, outcome measurement, adjustment for other prognostic factors, statistical analyses, and reporting. Each of the six domains was judged with high, moderate or low risk. A detailed description of the domains included in the tool and the judgment taken by the two reviewers is presented in Supplementary Material 7.

### Statistical analyses

In the pre-registration of the study, we registered a meta-analysis to investigate the predictive performance of the different prognostic factors. The goal was to meta-analyze groups of “similar” prognostic effect measures with a random effects approach to allow for unexplained heterogeneity across studies. However, after the data extraction, we found that data on prognostic factors of changes in memory test performance after memory training were too heterogeneous and too poorly reported to conduct a meta-analysis.

## Results

### Study selection

**Fig. 1:**
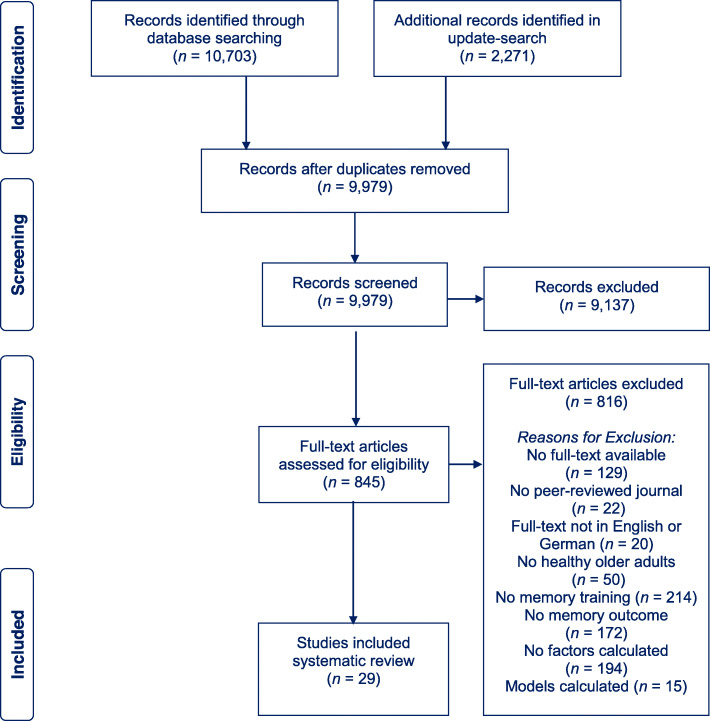
PRISMA diagram of the study selection process

The total number of retrieved references and the numbers of included and excluded studies with reasons for exclusions are documented in a flow chart as recommended in the PRISMA statement [16]. The PRISMA diagram in Fig. 1 illustrates the study selection process. Further, 10,703 studies were identified through the database search and by scanning the included studies in previously published systematic reviews and meta-analysis on memory training success in healthy older adults, *n* = 2271 studies were identified in an update search. After removing the duplicates, *n* = 9979 studies were screened. It was difficult to distinguish, from study abstracts alone, between prognostic factor finding studies and model development studies. We thus assessed 845 full-texts for eligibility. Finally, *n* = 29 studies were included in the present review. All studies were published in English.

### Data extraction

A main challenge was to distinguish between prognostic factor finding and model development studies, as the authors in general did not state their aim regarding prognostic factors or models. Therefore, we used full text interpretations to classify studies as prognostic factor finding or model development studies. Eight discrepancies were resolved after discussion with a third reviewer (NK) with experience in the field of prognostic research.

### Study characteristics

An overview of the main characteristics of the included studies is outlined in Table 1. Further information of the included studies is illustrated in Supplementary Tables 8 and 9.

**Table 1:** Study and participant characteristics of the included studies

Study	Sample				Training		Outcomes	Prognostic factors
Study design Initial sample size for the experimental group Dropout and reasons	Age (years, M, SD)	Sex		Education(years, M, SD)	Description of memory training—content and frequency	Total length of training in minutes	Definition and method of assessment Timing of outcome assessment	Definition and methods
		♂	♀					
**Pesce et al.** [20] Stratified randomized study *n* = 30 *n* = 29	70.40 (7.00)	14	15	9.60 (1.80)	Method of loci and general strategies. 24 weeks, 2 times a week for 1 h	2880	RAVLT, MMSE	Antioxidant levels assessed with the Biological Antioxidant potential Test; reactive oxygen metabolites derivative compounds assessed with the d-ROMs Test
**O'Hara et al.** [9] Non-randomized, non-controlled longitudinal study *n* = 531 *n* = 419 due to several reasons at 5-year follow-up	73.73(7.62)	34	78	15.56 (2.79)	Method of loci. 2 weeks, 5 times a week for 2 h	1200	Number of words correctly recalled, number of words correctly recalled in order. Assessed at baseline and 5-year follow-up measurement	Pre-training, gain scores following training, age, education, reported use of mnemonic at follow-up, type of pre-training (standard vs. comprehensive) and length of training.
**Mohs et al.** [21] RCT *n* = 68 *n* = n.a.	78.30 (7.40)	15	53	16.00 (2.70)	Structured memory training focusing on memory improvement and different strategies. Nine 90-min sessions	810	Verbal memory assessed with CVLT, non-verbal memory assessed with BFLT. Assessed 2 times at baseline, at post-test, 3 months and 6 months follow-up	Age, education, gender, subjective reported memory assessed with the MFI and the MFQ
**Kirchhoff et al**. [22] Non-randomized, non-controlled longitudinal study *n* = 16 *n* = 2 due to technical difficulties	72.00 (66–81)	7	7	14.70 (2.90)	Memory strategy training and practice. 2 training sessions	Missing information	Memory retrieval using Remember/Know/New recognition memory decisions Assessed at pre-training and post-training	Hippocampal activity
**Kirchhoff et al.** [23] Controlled trial *n* = 16 *n* = n.a.	71.9 (66–81)	8	8	14.8 (2.7)	Memory strategy training and practice 2 training sessions.	Missing information	Recognition memory using Remember/Know/New recognition memory decisions. Assessed at pre-training and post-training	Activity in prefrontal cortex, left lateral temporal cortex.
**Leahy et al.** [24, 25] RCT *n* = 22 *n* = 1	74.77 (6.57)	8	13	18.77 (2.62)	Memory specificity training to improve the specificity of older adults’ retrieval of autobiographical memories by providing systematic practice. 4 weeks, once a week for 60 min	240	Autobiographical memory specificity. Assessed at pre-test, post-test, and 3 months follow-up.	Memory specificity assessed with MEPS, functional limitations assessed with FLP, self-rated depression assessed with HADS, independence assessed with IADL
**Andrewes et al.** [26] RCT controlled for sex *n* = 20 *n* = 3	60–70 years	10	10	Some secondary schooling: *n* = 3 Secondary school + trade qualifications: *n* = 5 Complete secondary school: *n* = 6 Began tertiary school: *n* = 6	Memory handbook training for face-name and prospective memory areas; independently implemented at home 4 weeks, 30 min per session	Missing information	Improvement in: Face-name Test, Laboratory Prospective Memory Assessment, Everyday Prospective Memory Assessment. Assessed at pre-test, post-test and 4-month FU	RAVLT, Warrington Forced-Choice Recognition for Faces, BDI, NART, Mattis Dementia Rating Scale.
**Anschutz et al.** [27] Non-randomized, non-controlled longitudinal study *n* = 10 *n* = 1 due to severe illness	73.50 (n.a.)	2	7	10.70 (n.a.)	Method of loci No information on training duration and frequency	Missing information	Free-recall of two lists and recognition of two lists consisting of 12 nouns each. Assessed at pre-test and 34 months after finishing the training	Free-recall pre-test, free recall list 1, age
**Bissig and Lustig** [28] Non-randomized, non-controlled longitudinal study *n* = 19 *n* = 1 due to low accuracy of studied words	74.50 (6.10)	n.a.	n.a.	18.00 (3.30)	Modified recollection training procedure 2 weeks, 4 sessions per day at 7 days	Missing information	Ranking: participants were ranked by final lag level (lag between lure repetitions). Assessed and adapted individually during each training performance	Age, crystallized intelligence
**Bråthen et al.** [29] Controlled trial *n* = 126 *n* = 3	Old: 73.40 (3.00)	Old: 29	Old: 49	Old: 14.70 (2.90)	Learning and practicing the Method of loci technique aiming to improve episodic memory performance 10 weeks, once a week + 8 weekly online home assignments	Missing information	Memory improvement: change in correct written recall of word list consisting of 100 nouns. Assessed at pre-test and post-test	Cortical volume, hippocampal volume, ALFF, fALFF
**Brooks et al.** [8] RCT *n* = 224 Dropout not reported	68.58 (7.05)	n.a.	n.a.	15.33 (2.58)	Pre-training: imagery training, verbal elaboration and relaxation. Name-Face Mnemonic: three-step mnemonic Method of loci: method of loci for serial word recall. 2 weeks, 5 times a week for 120 min)	1200	Proper name recall task, word recall task (16 common words). Assessed at pre-test and post-test	Pretraining, pretest score, age, length of training, pretraining x length
**Clark et al.** [30] Multi-site RCT (ACTIVE) *n* = n.a. *n* = n.a.	No demographics separately for the memory training groups were reported.				Memory training focused on improving verbal episodic memory through instruction and practice in strategy use 6 weeks, 10 60-min sessions	600	HVLT, RAVL, RBMT. Measured at baseline, immediate post-training, 1-;2-, 3-; 5-, and 10-year FU	Obesity, determined from BMI (in kg/m^2^) computed from measured height and weight data obtained at baseline
**Clark et al.** [31] Multi-site RCT (ACTIVE) *n* = n.a. *n* = n.a.	No demographics separately for the memory training groups were reported.				Memory training focused on improving verbal episodic memory through instruction and practice in strategy use 6 weeks, 10 60-min sessions	600	Hopkins Verbal Learning Test, RAVL, RBMT. Measured at baseline, immediate post-training, 1-;2-, 3-; 5-, and 10-year FU	Education (self-reported as years of completed schooling)
**de Lange et al.**, [32] Controlled trial *n* = 76 *n* = 9 due to time constraints	73.60 (3.00)	25	51	15.00 (2.70)	Learning and practicing the Method of Loci technique aiming to improve episodic memory performance. 10 weeks, once a week + 8 weekly online home assignments	Missing information	Memory improvement: Word list recall	Interindividual variability in white matter microstructure
**de Lange et al.**, [33] Controlled trial *n* = 44 *n* = 0	73.30 (2.70)	21	23	15.70 (3.10)	Learning and practicing the Method of Loci technique aiming to improve episodic memory performance. 10 weeks, once a week + 8 weekly online home assignments	Missing information	Memory improvement: word list test (100 words)	White matter microstructure
**Tomaszewski Farias et al.** [34] Multi-site RCT (ACTIVE) *n* = n.a. *n* = n.a.	No demographics separately for the memory training groups were reported				Memory training focused on improving verbal episodic memory through instruction and practice in strategy use 6 weeks, 10 60-min sessions	600	Memory factor: Immediate recall HVLT, RAVLT, paragraph recall, RBMT	Instrumental activities of daily living, 18 questions of the Minimum Dataset Home Care scale
**Finkel and Yesavage** [35] Controlled trial *n* = 77 *n* = 16 due to illness (*n* = 5), frustration (*n* = 7), bad weather (*n* = 2), no reason (*n* = 1)	71.29 (6.31)	30%	70%	n.a.	Method of loci No information on training duration and frequency	Missing information	Memory improvement gain scores of a list of 16 common words recall	Age, education, MMSE score, depression score, neuroticism and extraversion scale of the NEO-PI
**Hampstead et al.** [36] RCT *n* = 12 *n* = 1 due to ongoing disease	73.20 (7.70)	n.a.	n.a.	16.10 (3.40)	Object Location Assignment encoding and retrieval with mnemonic strategy from a cognitive rehabilitation program 2 weeks, 5 sessions + 1 follow-up session one month later	Missing information	Modified change score of Object Location Assignment accuracy	Medial temporal lobe volumetrics (hippocampus, amygdala, inferior lateral ventricles), standardized neuropsychological measures (RBANS Delayed Memory Index, TMT B)
**Hill et al.** [37] Controlled trial *n* = 59 *n* = n.a.	67.80 (7.50)	n.a.	n.a.	5.80 (1.10)	Mnemonic training 2 weeks, twice a week for 120 min	1680	Recall performance in name-face recall	Rated confidence (perceived confidence in recalling the names of unfamiliar faces).
**Hill et al.** [38] Non-randomized, non-controlled longitudinal study *n* = 102 *n* = n.a.	75.40 (10.50)	32	70	n.a.	Name- and face and list-learning program using an imagery and judgment technique and method of loci method. 2 weeks, 7 times a week for 120 min	1680	Improvement in Name-Face recall, Improvement in List-Recall	MMSE.
**Leahy, Ridout, and Holland**, [24] RCT *n* = 20 *n* = 1 due to unrelated health problems	76.85 (5.27)	6	14	17.75 (2.65)	Memory flexibility program 4 weeks, once a week for 60 min	240	Autobiographical memory specificity in the AMT. Assessed at pre-test, post-test, and 3 month FU.	Baseline cognitive flexibility measured with the verbal fluency sub-score of ACE-III.
**López-Higes et al.** [39] RCT *n* = 50 *n* = 0	ApoE 4 carriers: 71.64 (5.72) Non-carriers: 71.68 (5.65)	n.a.	n.a.	n.a.	Memory training consisting of cognitive stimulation, memory concepts, management of forgetting everyday experiences, meta-memory training 3 months, 30 90-min sessions	2700	Logical Memory and Word List from WMS-III	Apolipoprotein E genotyping
**McDougall et al.**, [40] RCT *n* = 135 Loss to post-test: *n* = 8 Loss to FU: *n* = 12 Loss to end of study: *n* = 8	74.69 (5.74)	30	105	13.39 (3.90)	CBMEM-based intervention, based on the four components of self-efficacy theory 4 weeks, twice a week including 8 sessions and 4 booster sessions	720	HVLT-R, BVMT-R, RBMT. All outcome measures were administered at baseline, post-class (2 months after baseline), post-booster (6 months), post-classroom FU (14) and at the end of study (24 months)	Ethnicity, group assignment, time, education
**McDougall et al.**, [41] RCT *n* = 135 Loss to post-test: *n* = 8 Loss to FU: *n* = 12 Loss to end of study: *n* = 8	74.69 (5.74)	30	105	13.39 (3.90)	CBMEM-based intervention, based on the four components of self-efficacy theory 4 weeks, twice a week including 8 sessions and 4 booster sessions	720	Relative gains in HVLT-R, RBMT All outcome measures were administered at baseline, post-class (2 months after baseline), post-booster (6 months), post-classroom FU (14) and at the end of study (24 months)	Age, education, racial/ethnic group
**Neely & Bäckman** [42] RCT *n* = 23 *n* = n.a.	73.00 (4.20)	4	19	9.90 (3.10)	Encoding operations including interactive imagery and method of loci; attention training, relaxation training. Training was conducted in groups with 11–12 subjects, met twice a week for 5 consecutive weeks, each session lasted 1.5 h	900	Recall of concrete words, recall of objects, recall of subject-performed tasks, recall of abstract words Assessed at pre-test, post-test directly after training, 6 months FU	Pretest score for each dependent variable, MMSE score, age, years of education
**O’Hara et al.**, [43] Non-randomized, non-controlled longitudinal study *n* = 212 *n* = 113	74.00 (7.90)	68	32	15.50 (2.70)	Memory training was not further described. Missing information on duration and frequency.	Missing information	BVRT, Logical Memory Test, Associate Learning Test, List-learning test. Assessed at baseline and FU 4-5 years after memory training.	Apolipoprotein E genotyping.
**Park et al.** [7] RCT *n* = 39 *n* = n.a.	69.81 (4.90)	11	28	11.41 (4.31)	Multi-strategic memory training. 10 sessions once a week, each session lasted 1.5 h	900	Elderly verbal learning test of the EMS to assess verbal memory; Simple Rey Figure Test of the EMS to assess non-verbal memory. Assessed at pre-test and post-test (within 3 months after finishing the training)	All baseline values of the scores of neuropsychological tests, age, gender, years of education
**Rosi et al.**, [44] Non-randomized, non-controlled longitudinal study *n* = 44 *n* = n.a.	68.73 (6.05)	n.a.	n.a.	11.36 (3.50)	Memory training program. 6 weeks, once a week for 60 minutes.	360	Word list learning (memory practiced task), grocery list learning (memory non-practiced task), associative learning Assessed at pre-test and post-test.	Vocabulary test, Raven standard progressive matrices, listening span test, letter comparison, age
**Sandberg et al.** [45] Non-randomized, non-controlled longitudinal study *n* = 112 *n* = 18 due to various reasons	70.90 (6.70)	38	56	11.90 (3.70)	Mnemonic training was based on the Swedish version of the number-consonant mnemonic task 5 times, twice a week	600	Number recall. Assessed at pre-test, post-test and FU	Three measures of episodic memory (free recall of concrete nouns, free recall of abstract nouns, paired-associate recall), three measures of working memory (listening span, two versions of computation span), nine measures of processing speed, two measures of verbal knowledge, depression (ZSRDS), vocabulary

All reported values regarding sample size, dropouts, and sociodemographic variables only refer to the memory training groups. For the variables age (in years) and education (in years) means and standard deviations were displayed, when reported. Otherwise, ranges and/or absolute numbers are stated

*RAVLT* Rey Auditory Verbal Learning Task, *BDI* Beck Depression Inventory, *NART* National Adult Reading Test, *dROMs* reactive oxygen metabolites derivative compounds, *FU* follow-up, *ALFF* amplitude of low-frequency fluctuation, *fALFF* fractional amplitude of low-frequency fluctuation, *BMI* body mass index, *MMSE* Mini-Mental State Examination, *NEO*-*PI* NEO Personality Inventory, *RBANS* Repeatable Battery for the Assessment of Neuropsychological Status, *TMT B* Trial Making Test Version B, *AMT* Autobiographical Memory Task, *ACE*-*III* Addenbrooke’s Cognitive Examination-III, *CBMEM* Cognitive Behavioral Model of Everyday Memory, *HVLT*-*R* Hopkins Verbal Learning Test-Revised, *RBMT* Rivermead Behavioral Memory Test, *BVMT*-*R* Brief Visuospatial Memory Test revised, *EMS* Elderly Memory Disorder Scale, *BVRT* Revised Benton Visual Retention Test, *WMS*-*III* Wechsler Memory Scale III, *HVLT* Hopkins Verbal learning task, *MEPS* means end problem solving procedure, *FLP* functional limitation profile, *HADS* Hospital Anxiety and Depression Scale, *IADL* instrumental and basic activities of daily living, *CVLT* California Verbal Learning Test, *BFLT* Biber Figure Learning Test, *MFI* memory controllability inventory, *MFQ* Memory Functioning Questionnaire, *ZSRDS* Zung Self-Rating Depression Scale

Of the 29 studies included, we found that 15 studies used a randomized controlled design, whereas six studies only used a controlled design (Table 1). Furthermore, eight studies used a non-randomized, non-controlled longitudinal study design, which may be classified as a cohort study, as the defining characteristic of the cohort is the participants’ health status and attendance in memory training.

The sample sizes of the memory training intervention groups varied greatly between the studies, ranging from *n* = 10 participants [27] to *n* = 531 participants [9], with three studies not giving clear information on how many participants attended the memory training [30, 31, 34].

A detailed description of the different memory training interventions used (regarding content, length, and frequency) is displayed in Table 1. Seven studies stated that a strategy CT using the Method of Loci was conducted [8, 9, 27, 29, 32, 33, 35]. All other training programs differed in their content (e.g., learning and practicing of different memory strategies, memorizing grocery lists, psychoeducation about memory processes).

The mean age of the samples ranged from 67.8 years [37] to 78.3 years [21]. Yet, the samples were highly educated throughout the studies, ranging from a mean of 11.9 years [45] to a mean of 18.77 years of education [24, 25]. The mean score on the Mini-Mental State Examination (MMSE), which was assessed in 13 studies at baseline as an indicator for the participant’s global cognitive status at baseline, ranged from a mean of 25.9 points [30, 31] to 29.2 points [44]. In most studies, the samples consisted of more women than men, with an overall of 65.9% women and 34.1% men participating in the studies.

### Risk of bias

Regarding the reporting quality, Table 2 shows the risk of bias assessment according to the QUIPS tool [19] in all included studies. The table shows that there is important information lacking, especially regarding the domains study attrition, prognostic factor measurement, study confounding, and statistical analysis and reporting. Interestingly, the parameter outcome measurement was the only one in which all 29 studies provided a sufficient reporting and were rated as having a low risk of bias. A further important result was that statistical analysis and reporting was correctly accounted in eleven studies [9, 28–31, 33, 34, 40, 42, 44, 45]. Yet, all other studies which used correlation analysis or group comparisons as statistical methods to quantify prognostic factors were rated with a low reporting quality. This was also the case if no data was provided. Overall, the reporting quality was in part insufficient, and the studies in their entirety were difficult to comprehend, especially regarding the prognostic factor measurement, confounding and statistical analysis.

**Table 2:**
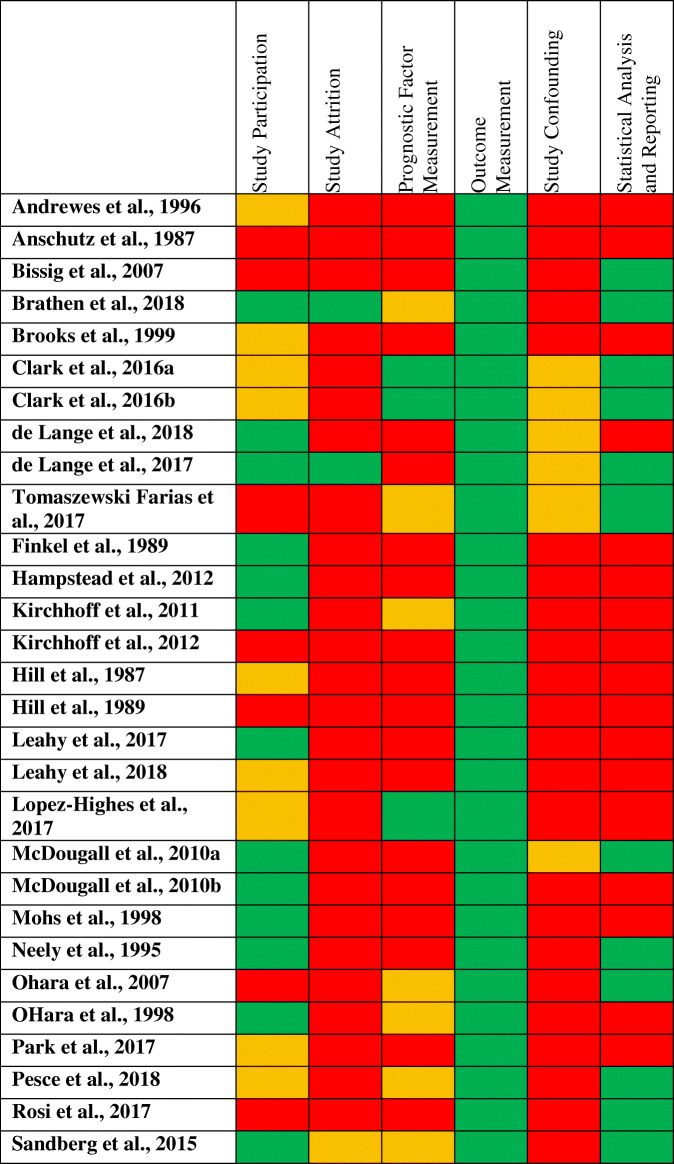
Risk of bias assessment

Red color indicates a high risk of bias, yellow color indicates a medium risk of bias, green color indicates a low risk of bias, assessed with the QUIPS tool [18]

### Outcomes and statistical outcome measures

In the present review, we investigated four outcomes: verbal short-term memory, verbal long-term memory, non-verbal short-term memory, and non-verbal long-term memory. Outcomes were well defined in all investigated studies. However, only five studies [7, 24, 25, 36, 42] reported that they blinded the outcome measurement. For a detailed overview of the different outcomes and their assessment, see Tables 3, 4, 5, and 6.

**Table 3:** Prognostic factors for training improvement in verbal short-term memory

Study	Test for outcome assessment	Dependent variable	Prognostic factor					
Multiple regression								
			Age	Education	Sex	Neuropsychology	Imaging	Others
**de Lange et al.**, [32]	Word list	Standardized residuals					White matter microstructure →	
**McDougall et al.** [40]	HVLT RBMT	Relative gains	↑			Pre-test score ↑		Ethnicity →
**Neely and Bäckman** [42]	Immediate recall of word list	Post-test scores	↓	↑		MMSE ↑ Pre-test score ↑ *		
**Rosi et al.** [44]	Immediate recall of word list	Post-test scores	↓			Pre-test ↑* Working memory ↓ Fluid ability ↓ Crystallized ability ↑* Processing speed ↑ Short-term memory ↓		
**Sandberg et al.** [45]	Number recall	Post-test scores	↓*			Episodic memory ↑* Processing speed ↓ Working memory ↑* Verbal knowledge ↑		
**Brooks et al.** [8]	Name recall	Post-test scores	↑*			Pre-test score*		Pretraining x mnemonic training →
Correlation analysis								
**Mohs et al.** [21]	HVLT	Post-test scores	→	→	→			Subjective reported memory →
**Kirchhoff, Anderson, Smith, Barch et al.**, [22]	Recognition memory decisions	Change score					Activity in frontal cortex ↑	
**Kirchhoff, Anderson, Smith et al.**, [22]	Recognition memory decisions	Change score					Activity in hippocampus ↑	
**Andrewes et al.** [26]	Face-name test	Change score				NART → RAVT → Warrington Forced Choice Recognition ↑		Depression → Mattis dementia scale →
**Bråthen et al.** [29]	Immediate recall of word list	n.a.					Hippocampal volume ↑* Amplitude of low frequency fluctuation ↓ Fractional amplitude of low frequency fluctuation ↓*	
**Finkel and Yesavage** [35]	Immediate recall of word list	Gain scores	x	x		MMSE x		Openness of experience ↑* Depression x Extraversion x Neuroticism x
**Hill et al.** [37]	Face-name recall	Standardized residual scores						Rated confidence ↑
**Hill et al.** [38]	Face-name recall	Performance changes				MMSE ↑		
Group comparisons (ANOVA, *t* test)								
**Clark, Xu, Callahan et al.**, [30]	HVLT RAVL RBMT	Relative mean improvement						Obesity ↓*
**Clark, Xu, Unverzagtet al.**, [31]	HVLT RAVL RBMT	Relative mean improvement		→				
**McDougall et al.** [40]	HVLT RBMT	n.a.		↓				Ethnicity (Blacks and Hispanics scored lower than Whites)
Mixed models								
**Tomaszewski Farias et al.** [34]	HVLT RAVL RBMT	Normalized residuals						Activities of daily living ↑
**López-Higes et al.** [39]	Word list recall Logical memory test	n.a.						Apolipoprotein E4 →
No clear reporting								
**Bissig and Lustig** [28]	Rank-test	n.a.	↓			Crystallized intelligence ↑		
**de Lange et al.**, [33]	Word list	Standardized residuals					White matter microstructure ↑	

Studies are sorted according to the statistical method used for obtaining the prognostic factors

*HVLT* Hopkins Verbal learning Task, *MMSE* Mini Mental State Examination, *NART* National Adult Reading Test, *RAVL* Rey Auditory Verbal Learning Test, *RBMT* Rivermead Behavioral Memory Test, ↑ the higher the prognostic factor, the higher the improvement/positive correlation, ↓ the lower the prognostic factor, the higher the improvement/negative correlation, → no direction of effect reported, * significant, x unclear reporting

**Table 4:** Prognostic factors for training improvement in verbal long-term memory

Study	Test for outcome assessment	Dependent variable	Prognostic factor					
Multiple regression								
			Age	Education	Sex	Neuropsychology	Imaging	Others
**O'Hara et al.** [9]	Number of words correctly recalled.	Post-test scores Pre-test and change scores were integrated in regression.	↓	↑		Gain scores following training ↑ *		Length of training (short vs. long) ↑ Reported use of mnemonic at follow-up ↑ * Type of pre-training (standard vs. comprehensive) ↓ Pre-training ↑ *
**Brooks et al.** [8]	Proper name recall task	Post-test scores	↑ *			Pre-test score →*		Pre-training * Length → Length of training → Pre-training →
**McDougall et al.** [40]	RBMT	Change score Relative gains from beginning to end of training	↑	x				Ethnical group x
**Park et al.** [7]	Elderly verbal learning test, delayed recall *However, results are reported for* “*cognitive function*” *as outcome measure*, *which is not clearly defined*	Change score Post-pre	→	↓*	→	Pre-test scores of neuropsychological tests (Digit Span Test, Spatial Span Test, Categorical Fluency Test, short version of Boston Naming test) →		
**Pesce et al.** [20]	RVLT	Change score Post-pre						Change in dROMs ↓ Change in BAP ↑
Correlation analysis								
**Leahy, Ridout, Mushtaq et al.**, [25]	Autobiographical memory specificity	Change score						Independence Depression Functional limitations Memory specificity
**Andrewes et al.** [26]	Laboratory Prospective Memory Assessment Everyday Prospective Memory Assessment	Change score				NART → Warrington Forced Choice Recognition → RAVT →		Mattis dementia scale → Depression →
**Anschutz et al.** [27]	Free recall of 2 lists Recognition of 2 lists	No clear reporting.	No clear reporting.					
**Hill et al.** [38]	Improvement in list recall	Change scores				MMSE ↑		
**Leahy, Ridout, and Holland**, [24]	Autobiographical memory specificity.	Change scores				Baseline cognitive flexibility ↑		
Group comparisons (ANOVA, *t* test)								
**McDougall et al.** [40]	RBMT	Pre-test and Post-test scores calculated in an ANOVA.	x	x				Ethnicity x
**O’Hara et al.**, [43]	List-learning test	Pre-test and Post-test scores calculated in an ANOVA.						Apolipoprotein E4 ↓
Mixed models								
/								

Studies are sorted according to the statistical method used for obtaining the prognostic factors

*ANOVA* analysis of variance, *MMSE* Mini Mental State Examination, *NART* National Adult Reading Test, *RAVL* Rey Auditory Verbal Learning Test, *RBMT* Rivermead behavioural memory test, *RVLT* Rey Auditory Verbal Learning Test, *dROMs* reactive oxygen metabolites derivative compounds, *BAP* antioxidant levels; ↑ the higher the prognostic factor, the higher the improvement/positive correlation; ↓ the lower the prognostic factor, the higher the improvement/negative correlation; → no direction of effect reported; * significant; x unclear reporting

**Table 5:** Prognostic factors for training improvement in non-verbal short-term memory

Study	Test for outcome assessment	Dependent variable	Investigated prognostic factor					
Multiple regression								
			Age	Education	Sex	Neuropsychology	Imaging	Others
**Park et al.**, [7]	Simple Rey Figure Test Immediate copy *However*, *results are reported for* “*cognitive function*” *as outcome measure*, *which is not clearly defined*	Change score Post-pre	→	↓*	→	Pre-test scores of neuropsychological tests (Digit Span Test, Spatial Span Test, Categorical Fluency Test, short version of Boston Naming test) →		
Correlation analysis								
**Mohs et al.** [21]	Biber Figure Learning Test	Post-test scores, Controlling for pre-test scores	→	→	→			Subjective reported memory →
Group comparisons (ANOVA, *t* test)								
/								
Mixed models								
/								

Studies are sorted according to the statistical method used for obtaining the prognostic factors. ↑ the higher the prognostic factor, the higher the improvement/positive correlation; ↓ the lower the prognostic factor, the higher the improvement/negative correlation; → no direction of effect reported; * significant; x unclear reporting

**Table 6:** Prognostic factors for training improvement in non-verbal long-term memory

Study	Test for outcome assessment	Dependent variable	Prognostic factor					
Multiple regression								
			Age	Education	Sex	Neuropsychology	Imaging	Others
**Park et al.** [7]	Simple Rey Figure Test Delayed Recall *However, results are reported for* “*cognitive function*” *as outcome measure*, *which is not clearly defined*	Change score Post-pre	→	↓*	→	Pre-test scores of neuropsychological tests (Digit Span Test, Spatial Span Test, Categorical Fluency Test, short version of Boston Naming test) →		
Correlation analysis								
**Hampstead et al.** [36]	Object Location Assignment accuracy	Modified change score Percentage of improvement relative to possible improvement after accounting for pre-test score				Trial Making Test B/A ↓ RBANS ↑	Amygdala volume ↑ Hippocampus volume ↑ Inferior lateral ventricles volume ↓	
Group comparisons (ANOVA, *t* test)								
**McDougall et al.** [40]	Brief Visuospatial Memory Test- Revised	ANOVA with pre- and post-test scores	→	→ *				Ethnicity—Hispanics and Blacks ↑* than Whites
**O’Hara et al.**, [43]	Revised Benton Visual Retention Test	ANOVA with pre- and post-test scores						Apolipoprotein E4 ↓*
Mixed models								
/								

Studies are sorted according to the statistical method used for obtaining the prognostic factors. *RBANS* Repeatable Battery for the Assessment of Neuropsychological Status; ↑ the higher the prognostic factor, the higher the improvement/positive correlation; ↓ the lower the prognostic factor, the higher the improvement/negative correlation; → no direction of effect reported; * significant; x unclear reporting

Twenty-one out of the 29 studies investigated verbal short-term memory as an outcome. Seven studies [29, 32, 33, 35, 39, 42, 44] used the immediate recall of a word list, which was the most frequently used test in this domain.

Twelve out of the 29 studies investigated verbal long-term memory. The delayed recall of a word-list test was the most frequently used test in four studies [9, 27, 38, 43].

Non-verbal short-term memory was only assessed in two out of 29 studies: one study used the immediate recall of the Simple Rey Figure test [7], the other used the Biber Figure Learning Test [21].

Four out of 29 studies assessed non-verbal long-term memory, all of them using different tests as outcome measures (see Table 6).

Prediction of more than one outcome was common, which may be due to their mostly exploratory aim.

Not only the used tests to measure the outcomes differed, but there was also substantial heterogeneity in the statistical outcome measures used. In total, eight studies used the post-test scores as the dependent variable for their calculations, whereas 18 studies used the change score (defined as post-pre scores) as the dependent variable for their prognostic factor calculation. Residual change scores were used as the dependent variable in only four studies, all of the defined as an outcome in the domain verbal short-term memory [32–34, 37]. For nine outcomes, there was no clear definition of the dependent outcome variable used for the prognostic factor measurement. None of the studies used percentile change scores as the dependent variable.

### Prognostic factors and statistical methods of prognostic factor analysis

There was no detailed description (e.g., a separate paragraph stating not only the name of the prognostic factor and method of measurement, but also blinding, and use in the statistical analysis (e.g., as a continuous or dichotomous factor)) of the candidate prognostic factors in most of the studies. Investigated prognostic factors include sociodemographic variables (i.e., age, sex, education, and ethnicity), neuropsychological test status at study entry in different domains, brain imaging measures, genetic variables (i.e., apolipoprotein E4), training characteristics, and personality traits (for a detailed overview, see Tables 3, 4, 5, and 6). The prognostic factor neuropsychological status at study entry, examined in 13 studies, was the most assessed prognostic factor [7, 8, 24–26, 28, 35, 38, 41–45], followed by age, which was assessed in eleven studies [7, 8, 21, 28, 35, 40–45]. Concerning other sociodemographic factors, education was tested as a prognostic factor in nine studies [7, 9, 21, 30, 31, 35, 40–42]; sex, however, was only investigated in two studies [7, 21] as a prognostic factor for changes in memory test performance after memory training. Six studies investigated different imaging factors [22, 23, 29, 32, 33, 36]. Other investigated prognostic factors were ethnicity [40, 41], subjective reported memory [21], depression [26, 35], “BIG 5” personality traits [35], self-rated confidence [37], obesity [30, 31], activities of daily living [24, 25, 34], apolipoprotein E 4 (a protein that is involved in the fat metabolism of the body and constitutes a risk factor for Alzheimer’s disease) [39, 43], biological antioxidant potential [20], and length of memory training [8, 9].

There were several different statistical methods used to calculate the impact of prognostic factors after memory training on memory outcomes. Eight studies calculated a multiple regression [7–9, 32, 41, 42, 44, 45] and two studies used a mixed model approach [34, 39]. Notably, 12 studies used correlation analysis to investigate prognostic factors [21–27, 29, 35–38]. Four studies [30, 31, 40, 43] used group comparisons (e.g., ANOVAs, *t* tests). In two studies [28, 33], there was no clear reporting on which statistical methods were used to determine the prognostic factors.

### Prognostic factors of change in memory test performance after memory training

One of the overall aims of the present systematic review was to systematize which prognostic factors are predictive for which of the four investigated memory outcomes. The results are summarized in Tables 3, 4, 5, and 6, structured according to the statistical method used for calculating the prognostic factors and the dependent outcome variables. There is a similar pattern that can be detected over all four outcome domains: The direction of the relationship between the prognostic factor and the memory outcome (the more of x/ the less of x) differ depending on which dependent variable is evaluated as the outcome measure. This finding is substantial for the interpretation of the current literature on prognostic factors of changes in memory test performance after memory training in healthy older adults.

The prognostic factor *age* was the factor that was investigated in most studies. Studies that used the post-test scores as the dependent outcome measure showed that participants with lower age showed greater improvements in memory test performance after training [9, 42, 44, 45] with only one exception [8]. However, it should be noted that the study of Brooks et al. [8] also integrated an interaction term in their analysis. In contrast, studies using the change score as the dependent variable found that participants with higher age benefit most from the training [41].

Of the six studies that assessed *education* as a prognostic factor, it was shown that studies which used the post-test score as the dependent variable showed that participants with a higher educational level benefit most from the training [9, 42], whereas the study which used the change score as the dependent variable [7] again showed the opposite results indicating that participants with a lower educational level show improvements in their memory test performance. All other studies did not report data on the prognostic factor.

*Sex* was only investigated in two studies as a prognostic factor for changes in memory test performance [7, 21]. Yet, both studies did not provide any data on the direction of the prognostic factor.

Studies which used the post-test score as the dependent variable in their calculation to assess *neuropsychological test scores at study entry* showed that participants with higher neuropsychological test scores at study entry significantly benefited more from the memory training [42, 44, 45]. All other studies did not report any significant results on the prognostic factor.

Six studies investigated *brain imaging* prognostic factors. Two studies showed that when using standardized residuals as the dependent variable, a higher integrity of *white matter microstructure* was predictive for improvements in memory performance [32, 33]. Furthermore, two studies using the change score showed that a higher *hippocampal volume* was predictive for improvements in memory performance [29, 36]. Furthermore, a higher *activity in the frontal cortex* [22, 23] and higher *activity in the hippocampus* were predictive for changes in memory performance when using the change score as the dependent variable in the calculations.

Other investigated prognostic factors were *ethnicity*, *subjective reported memory*, *depression*, *openness to experience*, *extraversion*, *neuroticism*, *obesity*, *activities of daily living*, *apolipoprotein E4*, *length of training*, *biological antioxidant potential*, and *independence*. The only significant results of these prognostic factors were regarding *openness to experience*, showing that a higher value on the openness to experience scale predicted higher changes in memory test performance when using the change score as the dependent variable [35], and regarding *obesity*, showing that lower obesity scores predict improvements in memory performance when using the change score as the dependent variable [30, 31].

## Discussion

This is the first systematic review that examines prognostic factors of changes in memory test performance after memory training in healthy older adults. The main findings are that (i) included studies used different types of dependent variables (change scores vs. post-test scores) when defining memory training success leading to contradictory results, and that (ii) age was the only variable investigated throughout most of the studies, showing that older adults showed improvements in memory test performance after training when using the change score as the dependent variable.

### Methodological considerations

The most important result is that the direction of the relationship between the prognostic factor and the memory outcome (the more of x/ the less of x) differ depending on which dependent variable is evaluated as the outcome measure. For example, this means that studies that used post-test scores as the dependent outcome measure showed that participants with lower age showed greater improvements in memory test performance after training [9, 42, 44, 45] with only one exception [8]. However, it should be noted that the study of Brooks et al. [8] also integrated an interaction term in their analysis. In contrast, studies using the change score as the dependent variable found that participants with higher age benefit most from the training [41]. This finding is substantial for the interpretation of the reported findings in the current literature on prognostic factors of changes in memory test performance after memory training in healthy older adults: Until now, different directions of prognostic factors have been reported, but the cause of these differences have remained unresolved. Discussed explanations in single studies included characteristics of the used memory training, measurement procedures and the investigated sample [45, 46]. The present systematic review suggests, however, that these heterogeneous findings can mainly be explained by the different statistical methods used for prediction analyses so far, and the different dependent outcome measures (post-test scores vs. change scores vs. residual scores). Therefore, when reading and interpreting prognostic factor data of memory training improvement, our systematic review shows that it is of outstanding importance to take a closer look on the dependent variable used to measure training improvement.

Our systematic review shows that the included studies not only used different dependent variables but also different statistical methods to calculate prognostic factors (e.g., linear regression models, correlation analyses, mixed models, and group comparisons). However, not all used methods are suitable to answer the question of who benefits from memory training. For example, correlation analysis do not imply causal relationship and are therefore not an appropriate tool for measuring predictive performance as prognosis is defined as estimating the risk of future outcomes in individuals based on different characteristics. Also, group comparisons (e.g., *t -* tests, ANOVAs) are not suitable for prognostic factor measurement, because they only show group differences. Yet, there are no clear recommendations regarding the “proper way” to calculate prognostic factors after memory training so far, even though it can be suggested that multiple regression analysis or structural equation models seem appropriate to answer the question of “who benefits” from training [47]. Smoleń et al. [47] suggest to use direct modeling of correlations between latent true measures and gain to investigate possible prognostic factors of changes in cognitive performances after CT.

Results of our review also show that investigated sample sizes in the included studies are often very small and that statistical power for the used calculations are lacking. It is important to note that the present review focuses on prognostic factors for memory performance after memory training instead of memory success after training.

### Identified prognostic factors for changes in memory performance

The only prognostic factor that has been measured in several studies investigating verbal short- and long-term memory is “age.” In studies which used the post-test score as the dependent variable [42, 44, 45], participants with younger age showed improvements after the memory training intervention, which may be explained by the magnification approach [48]. This account implies that participants who are already functioning at a high cognitive level can easily integrate new knowledge in already existing neuronal networks and can therefore profit faster and more easily from memory training. However, studies which use the change score as a dependent variable [41] show the opposite result: older participants benefited most from memory training. The latter result can be interpreted with the compensation hypothesis, stating that older participants may have more room for cognitive improvement [48]. This account implies that healthy older adults who are already functioning at optimal levels have less room for changes in memory training performance. When we look on the post-test performance, it is logical that younger participants who perform better at pretest also perform better after the training.

Further investigated prognostic factors include sociodemographic factors, neuropsychological test status at study entry in different domains, imaging measures, training characteristics, genetic variables (apolipoprotein E4), and personality traits. However, the reporting of most of the prognostic factors is insufficient so that only limited (or in some cases no) conclusions can be drawn from the data.

In one study, lower education was predictive for improvements in verbal long-term memory, non-verbal short-term memory, and non-verbal long-term memory when using the change score as a dependent variable [7]. These results might also be explained by the compensation hypothesis, showing that participants with less years of education show more room for cognitive improvement [48]. Yet, it is also important to keep in mind that the factor “education” might present more than just the years of schooling, but that it may be a proxy variable for socioeconomic status, early life factors, occupational health, or even the willingness to engage in lifelong learning or new activities [49–51]. All of these variables might affect the memory training performance and therefore additional variables should be taken into account in form of a prognostic model, to investigate the influence of years of education on training success while controlling for related covariates such as, e.g., socioeconomic status and cognitive reserve (which can be assessed with the help of questionnaires as the Lifetime of Experience Questionnaire [52]) or even also integrate these as possible further prognostic factors.

Regarding brain imaging factors, a higher hippocampal volume was a significant prognostic factor for improvements in memory performance after training in the domain verbal short-term memory [29]. However, it was not clearly reported which dependent variable was used in the study and therefore, clear conclusions of this result cannot be derived. In general, hippocampal-cortical connections are known to be critical for episodic memory functions [53], and it is known that the hippocampal volume is related to memory performance in older adults [54], and that memory training may enhance hippocampal activity [33]. Therefore, it seems plausible that a higher hippocampal volume constitutes a better “hardware” for memory plasticity. Further studies with a clear description and definition of the dependent variable used for measuring the prognostic effect of hippocampal volume on changes in memory test performance after memory training are needed to support this notion.

The apoE 4 allele, which is a well-known risk factor for Alzheimer disease [55] was a significant prognostic factor for improvements in memory test performance in non-verbal long-term memory. However, it was only assessed in a group comparison between carriers and non-carries of the allele, showing that non-carriers benefit more from training [43]. This finding is in line with a meta-analysis on the effects of apoE 4 on cognitive functions in non-impaired older adults [56], and a study on CT improvement of healthy older adults [46]. Interestingly, apoE and the apoE 4 human isoform both impair hippocampal neurogenesis and show therefore that apoE may influence hippocampal-related neurological diseases [57], showing a possible link between apoE 4 and hippocampal volume as prognostic factors of changes in memory test performance after memory training. However, further research is needed as only a limited number of studies have investigated the effects of apoE 4 on training performance so far.

The one study that studied obesity as a possible prognostic factor for changes in memory test performance after memory training using the relative change score as the dependent variable [30, 31] found that older adults with obesity had a significantly lower training effect on the memory score than adults with normal weight. This result may be indicative for a relationship between obesity and impaired neural plasticity. There is evidence of an effect of obesity on inflammation, and onward an effect of inflammation on cognitive function [58]. Besides, there are several studies showing that obesity or high-fat feeding are associated with deficits in learning, memory, and executive functions [59, 60]. Due to the fact that the World Health Organization reports that the number of obese people (body mass index, BMI > 30) and overweight (BMI > 25) is reaching epidemic proportions worldwide [61], obesity is an important prognostic factor to further investigate.

Taken together, regarding sociodemographic factors (e.g., age, education), it seems that more “vulnerable” groups show stronger changes in memory test performance after memory training, while regarding biological factors (including the prognostic factors hippocampal volume, apoE 4, and obesity), the opposite pattern occurs—possibly meaning that the latter factors may serve as the “hardware” that functions as a driver of plasticity. However, evidence is far too rare to identify consistent patterns in order to formulate a clear hypothesis and more research is needed.

A further result of our systematic review is that throughout the studies, the choice of investigated prognostic factors is highly heterogeneous and seems often rather arbitrary than theory-based. This may be due to the fact that prognostic factor research is often a study “add-on” or a secondary or tertiary aim instead of the primary research question, and therefore constitutes an exploratory research approach. Yet, selective reporting of outcomes (and prognostic factors) is often a risk [62] and without pre-registration of studies, it is impossible to detect whether outcomes were assessed but not reported. Unfortunately, until now, pre-registration of prediction research is not mandatory [63].

Summarized, most of the prognostic factors reported in this systematic review are still highly under-investigated. In order to ensure an individual, personalized medicine approach, however, it is of high importance to identify special prognostic factors for changes in memory test performance after memory training to provide the best fitting nonpharmacological intervention approach for the individual’s specific needs.

### Reporting quality in the included studies

As already mentioned, the fact that prognostic factor calculation was often used as an “add-on” may contribute to several methodological short-comings in some studies. Therefore, this may also explain the overall poor reporting quality of the included studies. Especially prognostic factors and their statistical measures were not adequately described in most of the studies included in this review. This result is in line with other systematic reviews on prognostic factors in other research populations (e.g., participants with low back pain, participants with cancer) showing many methodological shortcomings in the design and conduct of studies that address prognosis [64, 65]. This shows that there is an immediate need for adequate reporting in the area of prognostic factors for changes in memory test performance after memory training—and more generally. The methodological shortcomings in the primary literature limit conclusions about prognostic factors for memory training success.

### Limitations

When interpreting the results of this review, there are several limitations that have to be taken into account. First, it was difficult for the review authors to distinguish between prognostic factor and prognostic model studies, as the reporting was fairly poor in most studies. Most studies did not state whether their aim was to investigate a factor (the influence of one prognostic variable on the outcome), or a model (the influence of two or more prognostic variables and their interactions on the outcome). Further, the statistical methods were frequently not clearly reported so that in some cases, it was not possible to determine which prognostic variables were used in the final calculations. Therefore, a correct classification may not have been made in all included studies.

Furthermore, there was no scoring system regarding the assessment of the risk of bias tool QUIPS [19] to standardize the risk of bias assessment over other systematic reviews. However, a clear description of our risk of bias assessment procedure is provided in the Supplementary, so that traceability and replicability is provided.

In the present review, only studies published in English or German were included and therefore we may have missed studies published in other languages. As a further limitation, the present systematic review only focuses on memory outcomes after memory training, hereby disregarding other cognitive domains, as well as other non-cognitive outcomes (e.g., depression, quality of life, activities of daily living), and other single-domain (e.g., working memory training) and multi-domain CT, respectively. Further systematic reviews are needed to elaborate the knowledge on prognostic factors of CT success.

Unfortunately, we could not perform a meta-analysis on the investigated prognostic factors of memory training success as planned and described in the pre-registration of this systematic review (ID: CRD42019127479, https://www.crd.york.ac.uk/PROSPERO/). This had mainly two reasons: First, in most of the studies not enough or no statistical data at all was provided on the investigated prognostic factors, and second, the overall statistical reporting was too poor to extract the necessary details. Furthermore, due to the use of the different dependent variables, we could not integrate all available data in one single analysis without falsifying the results. When trying to calculate different analyses for the different dependent variables, we then had not enough data again to conduct the analyses.

### Strengths of this systematic review

A particular strength of the study is that it is the first review that focuses on prognostic factors for changes in memory test performance after memory training in healthy older adults. This systematic work was able to shed light on the reasons of inconsistent results of research regarding prognostic factors in the literature: they seem to be mainly due to different used methodological approaches.

A further strength is that the present review was conducted using Cochrane standards for systematic reviews. The present review further provides a differentiation among the different memory outcomes and a detailed reporting of the statistical methods of the included studies.

### Implications for further prognostic research

Yet, the results and conclusions regarding the statistical analysis of the prognostic factors for changes in memory test performance after memory training might also be transferred to other trainings and cognitive outcomes. As a clear recommendation, independent of the investigated non-pharmacological intervention and the investigated outcome, one should be aware of the used dependent variable and statistical methods to assess prognostic factors. We recommend the use of the change score as a dependent variable to answer the question “who benefits” from a nonpharmacological intervention and to use multiple regression analysis or structural equation models instead of correlation analysis and group comparisons.

## Conclusion

This present systematic review on prognostic factors of changes in memory test performance after memory training shows substantial short-comings in methodological reporting and statistical analyses and emphasizes the need of elaborated prognostic factor studies with large sample sizes, clear descriptions of prognostic factor and confounder measurement, and clear reporting standards. Furthermore, a special focus should clearly be on the use of the dependent variables used for prognostic factor calculation. Our systematic review also showed that most prognostic factors are still highly under-investigated. Prognostic factor research should not be an “add-on” to already existing studies, but should be a separate focus following clear reporting and conduction guidelines, as prognostic factor research is of high importance for aiding treatment and lifestyle decisions, improving individual dementia risk prediction, and providing new treatment options [6]. As a preliminary conclusion, regarding prognostic factors for changes in memory test performance after memory training, older adults seem to show greater improvements in memory test performance after memory training than younger adults.

## Supplementary information

**Additional file 1.** Table 1. The PRISMA for Abstracts Checklist. Table 2. The PRISMA checklist for systematic reviews. Table 3. Prognostic models for memory training success in healthy older adults, search strategy (CENTRAL). Table 4. Prognostic models for memory training success in healthy older adults, search strategy (Medline). Table 5. Prognostic models for memory training success in healthy older adults, search strategy (PsycInfo). Table 6. Prognostic models for memory training success in healthy older adults, search strategy (Web of Science Core Collection). Table 7. Risk of Bias Assessment using the QUIPS tool. Table 8. Outcomes, prognostic factors and details on analysis of the included studies. Note. Abbreviations: RAVLT: Rey Auditory Verbal Learning Task; BDI: Beck Depression Inventory; NART: National Adult Reading Test; ALFF: Amplitude of low-frequency fluctuation; fALFF: Fractional amplitude of low-frequency fluctuation; BMI: body mass index; MMSE: Mini-Mental Status Examination; RBANS: Repeatable Battery for the Assessment of Neuropsychological Status; HVLT-R: Hopkins Verbal Learning Test-Revised; AMT: Autobiographical Memory Task; RBMT: Rivermead Behavioral Memory Test; BVMT-R: Brief Visuospatial Memory Test revised; EMS: Elderly Memory Disorder Scale; BVRT: Revised Benton Visual Retention Test; MEPS: Means End Problem Solving Procedure; FLP: functional limitation profile; FU: Follow-up; HADS: Hospital Anxiety and Depression Scale; IADL: Instrumental and basic activities of daily living; NEO-PI:NEO Personality Inventory; ZSRDS: Zung Self-Rating Depression Scale; ACE-III: Addenbrooke’s Cognitive Examination-III. Table 9. Overview of study results. Abbreviations: AMT: Autobiographical Memory Task; BVRT: Revised Benton Visual Retention Test; MMSE: Mini-Mental Status Examination. NEO-PI:NEO Personality Inventory, MEPS: Means End Problem Solving Procedure; FLP: functional limitation.

## Data Availability

The datasets used and/or analyzed during the current study are available from the corresponding author on reasonable request.
