# Supplementary material for: Prognostic factors for change in memory test performance after memory training in healthy older adults: a systematic review and outline of statistical challenges
Source: Diagn Progn Res. 2020 May 21;4:7. doi: 10.1186/s41512-020-0071-8 (PMC7240921; doi:10.1186/s41512-020-0071-8)
Supplement: Supplementary file 1 — Additional file 1. Table 1. The PRISMA for Abstracts Checklist. Table 2. The PRISMA checklist for systematic reviews. Table 3. Prognostic models for memory training success in healthy older adults, search strategy (CENTRAL). Table 4. Prognostic models for memory training success in healthy older adults, search strategy (Medline). Table 5. Prognostic models for memory training success in healthy older adults, search strategy (PsycInfo). Table 6. Prognostic models for memory training success in healthy older adults, search strategy (Web of Science Core Collection). Table 7. Risk of Bias Assessment using the QUIPS tool. Table 8. Outcomes, prognostic factors and details on analysis of the included studies. Note. Abbreviations: RAVLT: Rey Auditory Verbal Learning Task; BDI: Beck Depression Inventory; NART: National Adult Reading Test; ALFF: Amplitude of low-frequency fluctuation; fALFF: Fractional amplitude of low-frequency fluctuation; BMI: body mass index; MMSE: Mini-Mental Status Examination; RBANS: Repeatable Battery for the Assessment of Neuropsychological Status; HVLT-R: Hopkins Verbal Learning Test-Revised; AMT: Autobiographical Memory Task; RBMT: Rivermead Behavioral Memory Test; BVMT-R: Brief Visuospatial Memory Test revised; EMS: Elderly Memory Disorder Scale; BVRT: Revised Benton Visual Retention Test; MEPS: Means End Problem Solving Procedure; FLP: functional limitation profile; FU: Follow-up; HADS: Hospital Anxiety and Depression Scale; IADL: Instrumental and basic activities of daily living; NEO-PI:NEO Personality Inventory; ZSRDS: Zung Self-Rating Depression Scale; ACE-III: Addenbrooke’s Cognitive Examination-III. Table 9. Overview of study results. Abbreviations: AMT: Autobiographical Memory Task; BVRT: Revised Benton Visual Retention Test; MMSE: Mini-Mental Status Examination. NEO-PI:NEO Personality Inventory, MEPS: Means End Problem Solving Procedure; FLP: functional limitation. [file 41512_2020_71_MOESM1_ESM.docx]

Supplementary Material:

Prognostic factors for change in memory test performance after memory training in healthy older adults: A systematic review and outline of statistical challenges

Table 1: The PRISMA for Abstracts Checklist

| **TITLE** | **CHECKLIST ITEM** | REPORTED ON PAGE # |
| --- | --- | --- |
| 1. Title: | Identify the report as a systematic review, meta-analysis, or both. | 1 |
| **BACKGROUND** |  |  |
| 2. Objectives: | The research question including components such as participants, interventions, comparators, and outcomes. | 2, l. 1 - 4 |
| **METHODS** |  |  |
| 3. Eligibility criteria: | Study and report characteristics used as criteria for inclusion. | 2, l.5 - 10 |
| 4. Information sources: | Key databases searched and search dates. | 2, l. 6 & 7 |
| 5. Risk of bias: | Methods of assessing risk of bias. | 2, l. 10 |
| **RESULTS** |  |  |
| 6. Included studies: | Number and type of included studies and participants and relevant characteristics of studies. | 2, l. 11 |
| 7. Synthesis of results: | Results for main outcomes (benefits and harms), preferably indicating the number of studies and participants for each. If meta-analysis was done, include summary measures and confidence intervals. | 2, l. 11 - 15 |
| 8. Description of the effect: | Direction of the effect (i.e. which group is favoured) and size of the effect in terms meaningful to clinicians and patients. | / |
| **DISCUSSION** |  |  |
| 9. Strengths and Limitations of evidence: | Brief summary of strengths and limitations of evidence (e.g. inconsistency, imprecision, indirectness, or risk of bias, other supporting or conflicting evidence) | 2, l. 18 - 20 |
| 10. Interpretation: | General interpretation of the results and important implications | 2, l.18 - 20 |
| **OTHER** |  |  |
| 11. Funding: | Primary source of funding for the review. | 1 |
| 12. Registration: | Registration number and registry name. | 2 |

Table 2: The PRISMA checklist for systematic reviews

| **Section/topic** | **#** | **Checklist item** | **Reported on page #** |
| --- | --- | --- | --- |
| **TITLE** | | |  |
| Title | 1 | Identify the report as a systematic review, meta-analysis, or both. | 1 |
| **ABSTRACT** | | |  |
| Structured summary | 2 | Provide a structured summary including, as applicable: background; objectives; data sources; study eligibility criteria, participants, and interventions; study appraisal and synthesis methods; results; limitations; conclusions and implications of key findings; systematic review registration number. | 2 |
| **INTRODUCTION** | | |  |
| Rationale | 3 | Describe the rationale for the review in the context of what is already known. | 3 - 6 |
| Objectives | 4 | Provide an explicit statement of questions being addressed with reference to participants, interventions, comparisons, outcomes, and study design (PICOS). | 6 |
| **METHODS** | | |  |
| Protocol and registration | 5 | Indicate if a review protocol exists, if and where it can be accessed (e.g., Web address), and, if available, provide registration information including registration number. | 6 |
| Eligibility criteria | 6 | Specify study characteristics (e.g., PICOS, length of follow-up) and report characteristics (e.g., years considered, language, publication status) used as criteria for eligibility, giving rationale. | 6 & 7 |
| Information sources | 7 | Describe all information sources (e.g., databases with dates of coverage, contact with study authors to identify additional studies) in the search and date last searched. | 6 |
| Search | 8 | Present full electronic search strategy for at least one database, including any limits used, such that it could be repeated. | Supplementary Material |
| Study selection | 9 | State the process for selecting studies (i.e., screening, eligibility, included in systematic review, and, if applicable, included in the meta-analysis). | 6 - 8 |
| Data collection process | 10 | Describe method of data extraction from reports (e.g., piloted forms, independently, in duplicate) and any processes for obtaining and confirming data from investigators. | 8 |
| Data items | 11 | List and define all variables for which data were sought (e.g., PICOS, funding sources) and any assumptions and simplifications made. | 8 |
| Risk of bias in individual studies | 12 | Describe methods used for assessing risk of bias of individual studies (including specification of whether this was done at the study or outcome level), and how this information is to be used in any data synthesis. | 8 |
| Summary measures | 13 | State the principal summary measures (e.g., risk ratio, difference in means). | 9 |
| Synthesis of results | 14 | Describe the methods of handling data and combining results of studies, if done, including measures of consistency (e.g., I^2^) for each meta-analysis. | / |

| **Section/topic** | **#** | **Checklist item** | **Reported on page #** |
| --- | --- | --- | --- |
| Risk of bias across studies | 15 | Specify any assessment of risk of bias that may affect the cumulative evidence (e.g., publication bias, selective reporting within studies). | 8 |
| Additional analyses | 16 | Describe methods of additional analyses (e.g., sensitivity or subgroup analyses, meta-regression), if done, indicating which were pre-specified. | / |
| **RESULTS** | | |  |
| Study selection | 17 | Give numbers of studies screened, assessed for eligibility, and included in the review, with reasons for exclusions at each stage, ideally with a flow diagram. | 9 |
| Study characteristics | 18 | For each study, present characteristics for which data were extracted (e.g., study size, PICOS, follow-up period) and provide the citations. | 10 - 12 |
| Risk of bias within studies | 19 | Present data on risk of bias of each study and, if available, any outcome level assessment (see item 12). | 12 & 13 |
| Results of individual studies | 20 | For all outcomes considered (benefits or harms), present, for each study: (a) simple summary data for each intervention group (b) effect estimates and confidence intervals, ideally with a forest plot. | / |
| Synthesis of results | 21 | Present results of each meta-analysis done, including confidence intervals and measures of consistency. | / |
| Risk of bias across studies | 22 | Present results of any assessment of risk of bias across studies (see Item 15). | 44 |
| Additional analysis | 23 | Give results of additional analyses, if done (e.g., sensitivity or subgroup analyses, meta-regression [see Item 16]). | / |
| **DISCUSSION** | | |  |
| Summary of evidence | 24 | Summarize the main findings including the strength of evidence for each main outcome; consider their relevance to key groups (e.g., healthcare providers, users, and policy makers). | 20 - 26 |
| Limitations | 25 | Discuss limitations at study and outcome level (e.g., risk of bias), and at review-level (e.g., incomplete retrieval of identified research, reporting bias). | 27 & 28 |
| Conclusions | 26 | Provide a general interpretation of the results in the context of other evidence, and implications for future research. | 29 |
| **FUNDING** | | |  |
| Funding | 27 | Describe sources of funding for the systematic review and other support (e.g., supply of data); role of funders for the systematic review. | 1; 30 |

*From:*  Moher D, Liberati A, Tetzlaff J, Altman DG, The PRISMA Group (2009). Preferred Reporting Items for Systematic Reviews and Meta-Analyses: The PRISMA Statement. PLoS Med 6(7): e1000097. doi:10.1371/journal.pmed1000097

Table 3: Prognostic models for memory training success in healthy older adults, search strategy **(CENTRAL)**

| 1. “healthy older adults”: ti, ab, kw |
| --- |
| 1. “healthy elderly”: ti, ab, kw |
| 1. MeSH descriptor: [Healthy aging] |
| 1. “older adults”: ti, ab, kw |
| 1. MeSH descriptor: [Aged] |
| 1. MeSH descriptor: [Aged, 80 and over”] |
| 1. “elderly individuals”: ti, ab, kw |
| 1. “cognitive aging”: ti, ab, kw |
| 1. “cognitive intervention”: ti, ab, kw |
| 1. “cognitive training”: ti, ab, kw |
| 1. “brain training”: ti, ab, kw |
| 1. “memory training”: ti, ab, kw |
| 1. “reasoning training”: ti, ab, kw |
| 1. “mnemonic training”: ti, ab, kw |
| 1. “training”: ti, ab, kw |
| 1. “intervention”: ti, ab, kw |
| 1. MeSH descriptor: [Memory] |
| 1. “memory”: ti, ab, kw |
| 1. {or #1-#8} |
| 1. {or #9-#16} |
| 1. #17 or #18 |
| 1. #19 and #20 and #21 |

Access: 01.08.2018; results: 738 (duplicates: 69)

Table 4: Prognostic models for memory training success in healthy older adults, search strategy **(Medline)**

1. “healthy older adults” [All fields]

2. “healthy elderly” [All fields]

3. “Healthy aging” [MeSh]

4. “older adults” [All fields]

5. “Aged” [Mesh:NoExp]

6. “Aged, 80 and over” [MeSh]

7. “elderly individuals” [All fields]

8. “cognitive aging” [All fields]

9. 1 OR 2 OR 3 OR 4 OR 5 OR 6 OR 7 OR 8

10. “cognitive intervention” [All fields]

11. “cognitive training” [All fields]

12. “brain training” [All fields]

13. “memory training” [All fields]

14. “reasoning training” [All fields]

15. “mnemonic training” [All fields]

16. “training” [All fields]

17. “intervention” [All fields]

18. 10 OR 11 OR 12 OR 13 OR 14 OR 15 OR 16 OR 17

19. “memory” [MeSh]

20. “memory” [all fields]

21. 19 OR 20

22. 9 AND 18 AND 21

Access: 01.08.2018; results: 3049

Table 5: Prognostic models for memory training success in healthy older adults, search strategy **(PsycInfo)**

1. exp memory/

2. exp Aging/

3. “healthy older adults” .mp.

4. “healthy elderly” .mp.

5. “older adults” .mp.

6. “cognitive aging” .mp.

7. “aged (attitudes toward)”/

8. “cognitive intervention” .mp.

9. “cognitive training” .mp.

10. “brain training” .mp.

11. “memory training” .mp.

12. “reasoning training” .mp.

13. “mnemonic training” .mp.

14. “training” .mp.

15. “intervention” .mp.

16. exp brain stimulation/ or exp brain training/

17. 8 or 9 or 10 or 11 or 12 or 13 or 14 or 15 or 16

18. 2 or 3 or 4 or 5 or 6 or 7

19. 1 and 17 and 18

Access: 01.08.2018; results: 739

Table 6: Prognostic models for memory training success in healthy older adults, search strategy **(Web of Science Core Collection)**

1. “healthy older adults” [All fields]

2. “healthy elderly” [All fields]

3. “Healthy aging” [MeSh]

4. “older adults” [All fields]

5. “Aged” [Mesh:NoExp]

6. “Aged, 80 and over” [MeSh]

7. “elderly individuals” [All fields]

8. “cognitive aging” [All fields]

9. 1 OR 2 OR 3 OR 4 OR 5 OR 6 OR 7 OR 8

10. “cognitive intervention” [All fields]

11. “cognitive training” [All fields]

12. “brain training” [All fields]

13. “memory training” [All fields]

14. “reasoning training” [All fields]

15. “mnemonic training” [All fields]

16. “training” [All fields]

17. “intervention” [All fields]

18. 10 OR 11 OR 12 OR 13 OR 14 OR 15 OR 16 OR 17

19. “memory” [MeSh]

20. “memory” [all fields]

21. 19 OR 20

22. 9 AND 18 AND 21

Access: 01.08.2018; results: 6187 (6 duplicates)

Table 7: Risk of Bias Assessment using the QUIPS tool

| Domains and subdomains assessed with the QUIPS tool | Issues to consider according to the QUIPS tool and judgment reasons of the review authors |
| --- | --- |
| **Study participation** | The domain was rated with high risk, if no inclusion or exclusion criteria were stated, or if more than two subdomains were rated as “high risk”. It was rated as “moderate risk”, if two domains were rated with a “high risk”. |
| - Source of target population | The source population or population of interest is adequately described. |
| - Method used to identify population | The sampling frame and recruitment are adequately described, including methods to identify the sample sufficient to limit potential bias |
| - Recruitment period | Period of recruitment was described. |
| - Place of recruitment | Place of recruitment (setting and geographic location) are adequately described |
| - Inclusion and exclusion criteria | Inclusion and exclusion criteria are adequately described (e.g., including explicit diagnostic criteria or “zero time” description). |
| - Adequate study participation | There is adequate participation in the study by eligible individuals |
| - Baseline characteristics | The baseline study sample (i.e., individuals entering the study) is adequately described for at least the variables age, sex, and education. |
| **Study Attrition** | The domain was rated with high risk, if more than two subdomains were rated as “high risk”. It was rated as “moderate risk”, if two domains were rated with a “high risk” or if either “Reasons for lost to follow-up” or “Outcome and prognostic factor information” was rated with a “high risk”. |
| - Proportion of baseline sample available for analysis | Response rate (i.e., proportion of study sample completing the study and providing outcome data) is adequate. |
| - Attempts to collect information on participants who dropped out | Attempts to collect information on participants who dropped out of the study are described. |
| - Reasons and potential impact of subjects lost to follow-up | Reasons for loss to follow-up are provided. |
| - Outcome and prognostic factor information on those lost to follow-up | Participants lost to follow-up are adequately described, for at least age, sex, and education. |
|  | There are no important differences between key characteristics (age, sex, education) and outcomes in participants who completed the study and those who did not. |
| **Prognostic Factor Measurement** | The domain was rated with high risk, if more than two subdomains were rated as “high risk” or if the subdomain “definition of prognostic factor” was rated as “high risk”. It was rated as “moderate risk”, if two domains were rated with a “high risk”. |
| - Definition of the PF | A clear definition or description of 'PF' is provided. |
| - Valid and Reliable Measurement of PF | Method of PF measurement is adequately valid and reliable to limit misclassification bias. |
|  | Continuous variables are reported or appropriate cut-points (i.e., not data-dependent) are used. |
| - Method and Setting of PF Measurement | The method and setting of measurement of PF is the same for all study participants. |
| - Proportion of data on PF available for analysis | Adequate proportion of the study sample has complete data for PF variable. |
| - Method used for missing data | Appropriate methods of imputation are used for missing 'PF' data. |
| **Outcome Measurement** | The domain was rated with “high risk”, if one subdomain was rated as “high risk”. |
| - Definition of the Outcome | A clear definition of outcome is provided, including duration of follow-up and level and extent of the outcome construct. |
| - Valid and Reliable Measurement of Outcome | The method of outcome measurement used is adequately valid and reliable to limit misclassification bias. |
| - Method and Setting of Outcome Measurement | The method and setting of outcome measurement is the same for all study participants. |
| **Study Confounding** | The domain was rated with “high risk”, if two or more subdomains were rated as “high risk”. The domain was rated with “medium risk” if one domain was rated with “high risk”. |
| - Important Confounders Measured | Important confounders, including treatments, are measured. |
| - Definition of the confounding factor | Clear definitions of the important confounders measured are provided. |
| - Valid and Reliable Measurement of Confounders | Measurement of all important confounders is adequately valid and reliable (e.g., may include relevant outside sources of information on measurement properties, also characteristics, such as blind measurement and limited reliance on recall). |
| - Method and Setting of Confounding Measurement | The method and setting of confounding measurement are the same for all study participants. |
| - Method used for missing data | Appropriate methods are used if imputation is used for missing confounder data |
| - Appropriate Accounting for Confounding | Important potential confounders are accounted for in the study design or in the analysis. |
| **Statistical Analysis and reporting** | The domain was rated with “high risk” if the subdomain “Presentation of analytical strategy” was rated as “high risk” or if more than one of the other subdomains was rated with “high risk”. The domain was rated with “medium risk” if one subdomain (except the first) was rated with “high risk”. |
| - Presentation of analytical strategy | There is sufficient presentation of data to assess the adequacy of the analysis. |
| - Model development strategy | The strategy for model building (i.e., inclusion of variables in the statistical model) is appropriate and is based on a conceptual framework or model. |
|  | The selected statistical model is adequate for the design of the study (e.g. regression model, mixed models). |
| - Reporting of results | There is no selective reporting of results. |

Table 8: Outcomes, prognostic factors and details on analysis of the included studies

| Study | Outcome | |  | Prognostic Factors | | | | Analysis | | |
| --- | --- | --- | --- | --- | --- | --- | --- | --- | --- | --- |
|  | Definition & number | Blinded? | Handling in analysis | Definition & number | Handling in analysis | Blinded? | Timing | Modelling method | Assumptions checked? | Selection of factors |
| O'Hara et al., 2007 | Number of words correctly recalled, Number of words correctly recalled in order. | n.a. | Post-test scores, baseline and gain scores were integrated in the regression. | Pre-training, gain scores following training, age, education, reported use of mnemonic at follow-up, type of pre-training (Standard vs. Comprehensive) and length of training. | Type of pre-training and length of training were categorized in 2 categories each. | n.a. | n.a. | Multiple regression | n.a. | n.a. |
| Mohs et al., 1998 | Verbal memory, non-verbal memory | n.a. | Post-test scores controlling for baseline scores. | Age, education, gender, subjective reported memory | Controlling for baseline scores. | n.a. | At participant presentation (2 baseline measures) | Partial correlation coefficients | n.a. | n.a. |
| Pesce et al., 2018 | Verbal long-term memory | n.a. | Change scores | Reactive Oxygen Metabolites derivative compounds; Antioxidant levels | Controlling for Age, Change in MMSE, Change in verbal short-term memory | n.a. | At participant presentation | Multiple regression | n.a. | n.a. |
| Kirchhoff et al., 2012 | Recognition memory using Remember/Know/New recognition memory decisions. | No | Training-related changes in remember hits | Hippocampal activity | n.a. | No | At participant presentation and post-test | Pearson Product Moment correlation | n.a. | n.a. |
| Kirchhoff et al., 2012 | Recognition memory using Remember/Know/New recognition memory decisions. | No | Change scores (post-training – pre-training) | Activity in prefrontal cortex and left lateral temporal cortex | Monte-Carlo multiple comparison correction. | No | At participant presentation and post-test | Pearson Product Moment correlation | n.a. | An automated algorithm identified activation peaks. |
| Leahy, Ridout, Mushtaq et al., 2018 | Autobiographical memory specificity | yes | Change score: difference between scores at 3 month follow-up and pre-training. | Memory specificity assessed with MEPS, functional limitations assessed with FLP, self-rated depression assessed with HADS, independence assessed with IADL | All pairs of variables were normally distributed. | yes | At participant presentation. | Pearson correlation | Yes, normal distribution with Shapiro-Wilk’s test | n.a. |
| Andrewes et al. 1996 | Face-name Test, Laboratory Prospective Memory Assessment, Everyday Prospective Memory Assessment, all *n* = 20 | n.a. | Change score: subtracting scores at the baseline from the first postintervention scores. | RAVLT, Warrington Forced-Choice Recognition for Faces, BDI, NART, Mattis Dementia Rating Scale, all *n* = 20 | n.a. | n.a. | At participant presentation | Pearson product-moment correlation | n.a. | n.a. |
| Anschutz et al., 1987 | Free-recall of two lists and recognition of two lists consisting of 12 nouns each.  *n* = 9 at FU | n.a. | n.a. | No clear reporting.  Free-recall Pre-test, free recall list 1, age | n.a. | n.a. | Baseline values at participant presentation, age not reported. | Rank order correlations | n.a. | n.a. |
| Bissig & Lustig, 2007 | Rank | n.a. | n.a. | Age  Cristallised intelligence assessed with the Extended Range Vocabulary Test | n.a | n.a | At participant presentation | n.a | n.a. | n.a. |
| Brathen et al., 2018 | Memory improvement | n.a. | n.a. | Memory improvement: baseline hippocampal volume, ALFF, fALFF | ALFF/fALFF scores were not-normally distributed | n.a. | At participant presentation | Partial Spearman correlations/  Multiple regression | Normal distributions checked (Shapiro-Wilk test) | Theoretically based |
| Brooks et al., 1999 | Name recall post-score,  Word recall post-score | n.a. | Post-test scores were used as dependent variables | Name recall: Pretraining, pretest score, age  Word recall:  Pretraining, pretest score, age, length of training, pretraining x length interaction | n.a. | n.a. | At participant presentation | Name recall: Multiple regression  Word recall: Multiple regression | n.a. | n.a. |
| Clark, Xu, Callahan et al., 2016 | Hopkins Verbal Learning Test, RAVLT, RBMT | n.a. | Mean improvement from baseline for subjects in a training arm relative to the mean improvement for subjects in the control arm | BMI (in kg/m²). 3 classes were created according to the World Health Organization Criteria:  Normal: 16.5 – 24.9  Overweight: 25.0 – 29.9  Obesity: ≥ 30.0 | BMI was treated as a categorical variable. | Calculation was done after the experiment took place. | Data was obtained at participant presentation. | Repeated-measures mixed effect model | Tests for normal distributions | Theoretically based |
| Clark, Xu, Unverzagt et al., 2016 | Hopkins Verbal Learning Test, RAVLT, RBMT | n.a. | Mean improvement from baseline for subjects in a training arm relative to the mean improvement for subjects in the control arm | Education (self-reported as years of completed schooling) categorized in 4 categories:   1. Did not complete upper secondary education 2. Completed upper secondary education 3. Completed some tertiary education 4. Completed tertiary education | Education treated as categorical variable. | n.a. | At participant presentation. | Repeated-measures mixed effect model | Tests for normal distributions | Theoretically based |
| Lange et al., (2016) | Improvement in Digit Span Backwards | n.a. | Standardized residuals for each participant were used as the measure of change in memory performance. | Interindividual variability in white matter microstructure | n.a. | n.a. | At participant presentation | Linear regression model | n.a. | n.a. |
| Lange et al., 2017 | Memory Improvement in word list | Not blinded. | Standardized residuals. | Interindividual variability in white matter microstructure | n.a. | n.a. | Assessed at pre- and post-test. | n.a. | n.a. | n.a. |
| Tomaszewski Farias et al., 2018 | Memory change | n.a. | Normalized residuals | Instrumental activities of daily living, 18 questions of the Minimum Dataset Home Care scale | Single factor, each item was collapsed into a dichotomous rating of “any difficulty” and “no difficulty” | n.a. | Assessed at the 2,3,5 and 10 year follow-up test | Latent change score models | Yes, Item-level fit assessed using normalized residuals between model-estimated correlations and sample correlations for each pairwise correlation in a factor | Theoretically based |
| Finkel & Yesavage (1989) | Gain scores of a list of 16 common words recall | n.a. | Gain scores. | Age, Education, MMSE score, depression score, Neuroticism and Extraversion scale and Openess to Experience (*n* = 59) of the NEO-PI | n.a. | n.a. | At participant presentation. | Correlation analysis, no further information | n.a. | n.a. |
| Hampstead et al., (2012) | Modified change score of Treatment efficacy (percent of improvement relative to that possible after accounting for pre-training performance) of Object Location Assignment accuracy | yes | Modified change score of Treatment efficacy (percent of improvement relative to that possible after accounting for pre-training performance) | RBANS (*n* = 11)  Trails B/A (*n* = 11)  Inferior lateral ventricles (*n* = 11)  Hippocampus (*n* = 11)  Amygdala (*n* = 11) | Trails B/A ratio was calculated using demographically corrected T-scores | Neuropsychological assessment was blinded. | At participant presentation | Correlation analyses (Spearman’s rho) | n.a. | Theoretically based |
| Hill et al., 1987 | Name-face recall | n.a. | Standardized residual scores | Confidence in recalling unfamiliar faces | n.a. | n.a. | At participant presentation. | Correlation analysis | n.a. | Theoretically based |
| Hill, Yesavage, Sheikh, & Friedman, 1989 | Name-face recall;  List-learning recall (memorizing wordlist in original order – 16 words) | n.a. | Performance changes. | Mini-Mental State Examination score | n.a. | n.a. | n.a. | Correlation analysis | n.a. | Theoretically based |
| Leahy et al. (2018) | Change score for AMT Positive-ps, AMT Negative-ps total AMT-pS | n.a. | Changes scores were calculated by taking the difference between scores at 3-months and pretraining. | Baseline cognitive flexibility measured with the verbal fluency sub-score of ACE-III | n.a. | n.a. | At participant presentation | Correlation analysis, not further defined | n.a. | Theoretically based |
| López-Higes et al., 2017 | Word List and Logical Memory performance | n.a. | Pre- and Postscores | Apolipoprotein 4 genotyping | n.a. | n.a. | n.a. | Mixed linear model | n.a. | Theoretically based |
| McDougall et al., 2010a | HVLT-R, BVMT-R, RBMT. | n.a. | n.a. | Ethnicity, group assignment, time, and education. | n.a. | n.a. | At participant presentation | Multivariate approach to repeated measure analysis. | n.a. | n.a. |
| McDougall et al., 2010b | Relative gains in HVLT-R, RBMT. | n.a. | Relative gains from beginning to end of classes and from beginning to end of the 26-month study. | Age, Education, ethnicity/race | n.a. | n.a. | At participant presentation | Hierarchical linear models | n.a. | n.a. |
| Neely & Bäckman, 1995 | Recall of concrete words, recall of objects, recall of subject-performed tasks, and recall of abstract words. | Yes | Post-test scores with pre-test as control. | Prestest score for each dependent variable, MMSE score, age, and years of education. | Predictor variables were entered in 2 steps: first, the pretest score for each dependent measures, second all other variables added simultaneously | Neuropsychological assessment was blinded. | At participant presentation. | Hierarchical regression analysis with post-test scores as dependent variable. | n.a. | Theoretically based |
| O'Hara et al., 1998 | BVRT, Logical Memory Test, Associate Learning Test, List-learning test. | n.a. | Pre-test and Post-test scores. | Apolipoprotein 4 genotyping. | n.a. | n.a. | Genotyping was determined at follow-up. | Analysis of variance | n.a. | Theoretically based |
| Park et al. (2018) | Elderly verbal learning test of the EMS to assess verbal memory; Simple Rey Figure Test of the Elderly Memory Disorder Scale to assess non-verbal memory. | yes | Change score, post-score – pre.score | Pretest scores of all investigated neuropsychological tests, age, gender, years of education | Standardization to the respective z-scores; but also relative performance change calculated as post-score minus pre-score | Neuropsychological assessment was blinded. | At participant presentation. | Stepwise multiple regression | n.a. | Theoretically based |
| Rosi et al. (2017) | Word list learning (memory practiced task), grocery list learning (memory non-practiced task), and associative learning.  Assessed at pre-test and post-test. | n.a. | Memory score post-test score as dependent variable. | Vocabulary test, Raven standard progressive matrices, listening span test, letter comparison, age. | Predictor variables were entered in 2 steps: first, the pretest score for each dependent measures, second all other variables added simultaneously | n.a. | At participant presentation | Stepwise regression | n.a. | Theoretically based |
| Sandberg et al. (2015) | Number recall, assessed at pre-test, post-test and FU. | n.a. | Post-test scores, controlling for pre-test performance in the first step of the regression. | Three measures of episodic memory (free recall of concrete nouns, free recall of abstract nouns, paired-associate recall), three measures of working memory (listening span, two versions of computation span), nine measures of processing speed, two measures of verbal knowledge, depression (ZSRDS), vocabulary | Averaged z-scores were used as predictors. Predictor variables were entered in 2 steps: first, the pretest score for each dependent measures, second all other variables added simultaneously | n.a. | At participant presentation | Multiple regression analysis | n.a. | Theoretically based |

Note. Abbreviations: RAVLT: Rey Auditory Verbal Learning Task; BDI: Beck Depression Inventory; NART: National Adult Reading Test; ALFF: Amplitude of low-frequency fluctuation; fALFF: Fractional amplitude of low-frequency fluctuation; BMI: body mass index; MMSE: Mini-Mental Status Examination; RBANS: Repeatable Battery for the Assessment of Neuropsychological Status; HVLT-R: Hopkins Verbal Learning Test-Revised; AMT: Autobiographical Memory Task; RBMT: Rivermead Behavioral Memory Test; BVMT-R: Brief Visuospatial Memory Test revised; EMS: Elderly Memory Disorder Scale; BVRT: Revised Benton Visual Retention Test; MEPS: Means End Problem Solving Procedure; FLP: functional limitation profile; FU: Follow-up; HADS: Hospital Anxiety and Depression Scale; IADL: Instrumental and basic activities of daily living; NEO-PI:NEO Personality Inventory; ZSRDS: Zung Self-Rating Depression Scale; ACE-III: Addenbrooke’s Cognitive Examination-III.

Table 9: Overview of study results

| Study | Unadjusted Results  95% CI | Adjusted Results  95% CI | Set of adjusted factors used | Non-linear relation? | Modelling assumptions? |
| --- | --- | --- | --- | --- | --- |
| O'Hara et al., 2007 | Number of words correctly recalled:  A statistically significant proportion of variance was accounted for by pre-training, gain scores and use of the mnemonic at follow-up, but not by age, years of education, type or length of pre-training.  Number of words correctly recalled in order:  A statistically significant proportion of the variance was accounted for by pre-training and gain scores, but not by age, education, mnemonic use at follow-up, type of length of training. | / | No adjustment | n.a. | n.a. |
| Mohs et al., 1998 | No significant correlations were found between the objective and subjective measures at any post-intervention session visit controlling for the baseline scores. None of the demographic variables (age, education, gender) were significant predictors of change on the CVLT or subjective measures. | / | No adjustment. | n.a. | n.a. |
| Pesce et al., 2018 | / | The change in antioxidant levels was the best predictor of the variance in long-term verbal memory. | Age, Change in MMSE, Change in short-term verbal memory. | n.a. | n.a. |
| Kirchhoff et al., 2012 | A positive correlation was found between training-related changes in Remember Hits and activity for old words in the left hippocampus. | / | No adjustment. | n.a. | n.a. |
| Kirchhoff et al., 2012) | Strong positive correlations were found between training related changes in recognition and brain activity during encoding in the medial superior frontal, left middle frontal, left dorsal posterior inferior frontal, left ventral posterior inferior frontal, left anterior inferior frontal, left middle/superior temporal and right postcentral gyri. | / | No adjustment. | n.a. | n.a. |
| Leahy, Ridout, Mushtaq et al., 2018) | Change in autobiographical memory specificity was positively correlated with change in MEPS number of means, r = .53, P = .01. IADL, HADS and FLP were not significantly correlated. | / | No adjustment. | n.a. | Correlations also checked in the control group. |
| Andrewes et al. (1996) | No significant relationships were found.  No statistical values were provided. | / | No adjustments | n.a. | n.a. |
| Anschutz et al., (1987) | Age was negatively related to List 2 Free recall (*Rho* = -.81), *p* <.01). No other correlations were significant.  No other statistical values were reported. | / | No adjustments | n.a. | n.a. |
| Bissig & Lustig, (2007) | Age was negatively related to Rank, *ß* = .54; crystallized intelligence was positively related to rank, *ß* = -.51 | / | No adjustments | n.a. | n.a. |
| Brathen et al., (2018) | Hippocampal volume: Spearmans *rho* =.28, *p* = .04 in older adults, Spearmans *rho* =.15, *p* = .33 in younger adults.  fALFF: Spearmans *rho* =-.30, *p* =.03 in older adults, *rho* =.15, *p* =.32 in younger adults.  ALFF: *rho* =-.20, *p* = .09 in older, *rho* = -.03, *p* = .83 in younger adults.  Multiple regression with fALFF (*ß* = -.03) and hippocampal volume (*ß* = 0.27) as predictors. | / | No adjustments in multiple regression | No, correlations checked | n.a. |
| Brooks et al., (1999) | Analysis 1 Name recall:  Pretest score: *F*(1,219) = 58.42, *p* =.01; age: *F*(1,219) = 10.80, *p* =.01; pretraining x mnemonic training: **F**(1,219) < 1, n.s.  Analysis 2 Word recall:  Pretest score: *F*(1,217) = 85.41, *p* =.01; Age: *F*(1,217) = 10.63, *p* =.01; age x mnemonic training, *F*(1,217) = 5.33, *p* = .02; no significant effect in age x pretraining and age x pretraining x mnemonic training. | / | No adjustment | n.a. | n.a. |
| Clark, Xu, Callahan et al., 2016) | Older adults with obesity had a statistically lower training effect on memory composite score compared with adults with normal weight at post-training that carried through to 1 year (*p* = 0.02). | Older adults with obesity had a statistically lower training effect on memory composite score compared with adults with normal weight at post-training that carried through to 1 year (*p* = 0.01). | Age, female sex, minority race, married, Body mass index, current smoker, alcohol use, Short Form 36 Physical Functioning, depressions core, hypertension. Type 2 diabetes, stroke, congestive heart failure, ischemic heart disease, high cholesterol. | Non-linear relation of time variable was checked; therefore time was entered as a categorical variable. | Sensitivity Analysis were conducted with MMSE score and visual acuity as additional covariates and change in BMI to 2,3,5 and10 year FU. |
| Clark, Xu, Unverzagt et al., 2016) | / | There were no statistically significant differences on memory composite scores by educational attainment at immediate post-training or 1 year post-training. | Age, female sex, minority race, married, Body mass index, current smoker, alcohol use, Short Form 36 Physical Functioning, depressions core, hypertension. Type 2 diabetes, stroke , congestive heart failure, ischemic heart disease, high cholesterol, myocardial infarction, MMSE score, visual acuity, field site | Non-linear relation of time variable was checked; therefore time was entered as a categorical variable. | Sensitivity Analysis were conducted and estimated in a model with education as a continuous variable. |
| Lange et al., (2016) | No relationship was found between WM integrity at baseline and training improvement. | / | n.a. | n.a. | n.a. |
| Lange et al., (2017) | / | A positive relationship was found between change in FA and memory improvement in older adults in 12.87% of the voxels. Changes in MD, RD, and AD correlated negatively with memory improvement. | Age, sex, motion. | n.a. | n.a. |
| Tomaszewski Farias et al., 2018 | Preceding individual differences in IADLs appear to be driving subsequent memory change (RMSEA = 0.068, CFI = 0.932) | / | No adjustment | A linear relationship seems to best describe the relation | Checked, X² = 16.97, df = 1, CFI = 0.932 |
| Finkel & Yesavage (1989) | No significant correlations were found between improvement and any of the background variables (age, education, MMSE score, depression score) or with the Neuroticism or Extraversion scales of the NEO-PI. Openness of Experience of NEO-PI correlated with gain in recall scores (r =.293, n = 59, p <. 05). | / | No adjustment | n.a. | n.a. |
| Hampstead et al., (2012) | Spearman’s rho correlations with p-values in parentheses:  RBANS = .44 (.18)  Trails B/A = -.02 (.96)  Interior lateral ventricles = -.40 (.33)  Hippocampus = .02 (.97)  Amygdala = .04 (.93) | / | No adjustment | n.a. | n.a. |
| Hill et al., 1987) | The standardized residual scores from the regression of recall performance on rated confidence were correlated for the mnemonic subjects (*r* = .49, n = 59, *SEM* = .86). | / | No adjustment. | n.a. | Tested against slopes of the residual scores for both groups. |
| Hill et al. (1989) | Correlation of .30 between MMSE score and name-face recall improvement after the second week. Correlation of .27 between MMSE and list-learning improvement. | Results remained significant after adjustment, no data provided. | Regression analyses with preliminary score and age as adjusting variables. | n.a. | n.a. |
| Leahy et al. (2018) | Adjacency (combined) cognitive flexibility score at pre-training was correlated with:  Overall change in AMT-ps: r = 0.34, *p* =.04; change in AMT Negative-pS: r = 0.38, *p* = .02, change in AMT Positive-pS: n.s. | / | No adjustment | n.a. | n.a. |
| López-Higes et al., 2017 | There was no effect of apoE 4 on memory performance. | / | n.a. | n.a. | n.a. |
| McDougall et al., 2010a | On verbal memory, less educated Black and Hispanic participants scored lower than Whites.  Hispanics and Blacks performed better than Whites on visual memory. Everyday memory performance: Older, less educated participants and Hispanic and Black participants scored lower. | / | No adjustment | n.a. | n.a. |
| McDougall et al., 2010b | / | End of Study: Visual memory: Hispanic and Black participants gained more than Whites, participants with less education gained more.  Everyday memory: higher baseline and older participants gained more.  Self-reported measures: younger participants reported a greater reduction. | Age, education, racial/ethnic group, number of classes attended. | Analysis allows for a non-linear trajectory. | n.a. |
| Neely & Bäckman, 1995) | The pretest score for each of the dependent measures was a significant predictor of posttest performance. All other predictors did not increase significantly the proportion of explained variance. | / | No adjustment. | n.a. | n.a. |
| O'Hara et al., 1998 | A main effect of time was observed in the MMSE, BVRT, and Quick vocabulary test, with lower performance scores observed at follow-up. A significant improvement in performance was observed on the Logical Memory Test. | / | No adjustment. | n.a. | n.a. |
| Park et al. (2018) | Lower years of education (*ß* = -0.381, *p* = 0.02) predicted the performance change in cognitive function, no other predictors were significant. | / | No adjustment. | n.a. | n.a. |
| Rosi et al. (2017) | Word list learning: higher baseline performance explained a significant percentage of variance (*R²* = .33). Crystallized ability emerged as a significant predictor.  Grocery list learning: only higher baseline performance significant (*R²* = .38).  Associative learning: Higher baseline performance and processing speed were significant predictors. | / | No adjustment. | n.a. | n.a. |
| Sandberg et al. (2015) | Baseline level episodic memory, speed of processing and working memory were significant predictors of the residualised gains in number recall. | / | No adjustment. | n.a. | n.a. |

*Note.* Abbreviations: AMT: Autobiographical Memory Task; BVRT: Revised Benton Visual Retention Test; MMSE: Mini-Mental Status Examination. NEO-PI:NEO Personality Inventory; MEPS: Means End Problem Solving Procedure; FLP: functional limitation profile; HADS: Hospital Anxiety and Depression Scale; IADL: Instrumental and basic activities of daily living, CVLT: California Verbal Learning Test, RBANS: Repeatable Battery for the Assessment of Neuropsychological Status; ALFF: Amplitude of low-frequency fluctuation; fALFF: Fractional amplitude of low-frequency fluctuation; BMI: body mass index; FU: follow-up; WM: working memory;
